# Expanding the field of view – a simple approach for interactive visualisation of electron microscopy data

**DOI:** 10.1242/jcs.262198

**Published:** 2024-10-23

**Authors:** Jens Wohlmann

**Affiliations:** Department of Biosciences, University of Oslo, Blindernveien 31, PO Box 1041, 0316 Oslo, Norway

**Keywords:** Electron microscopy, Data communication, Interactive visualization, Reference space, Teaching

## Abstract

The unparalleled resolving power of electron microscopy is both a blessing and a curse. At 30,000× magnification, 1 µm corresponds to 3 cm in the image and the field of view is only a few micrometres or less, resulting in an inevitable reduction in the spatial data available in an image. Consequently, the gain in resolution is at the cost of loss of the contextual ‘reference space’, which is crucial for understanding the embedded structures of interest. This problem is particularly pronounced in immunoelectron microscopy, where the detection of a gold particle is crucial for the localisation of specific molecules. The common solution of presenting high-magnification and overview images side by side often insufficiently represents the cellular environment. To address these limitations, we propose here an interactive visualization strategy inspired by digital maps and GPS modules which enables seamless transitions between different magnifications by dynamically linking virtual low magnification overview images with primary high-resolution data. By enabling dynamic browsing, it offers the potential for a deeper understanding of cellular landscapes leading to more comprehensive analysis of the primary ultrastructural data.

## Teaser

**Summary:** A simple and easy to implement interactive visualisation for EM data, inspired by digital maps, which compensates for the loss of field of view at high magnifications and improves data communication and analysis.

## INTRODUCTION

Electron microscopy (EM) using thin sections has long been the gold standard for the study of cellular structures due to its unmatched resolving power and the ability to decipher the entire ultrastructural context of the cell. The detailed images obtained with EM enable the visualisation of the intricate ultrastructural features of cells and tissues and the localisation of molecules relative to this background by immunoelectron microscopy (immuno-EM).

### Problems

**Fig. 1.**
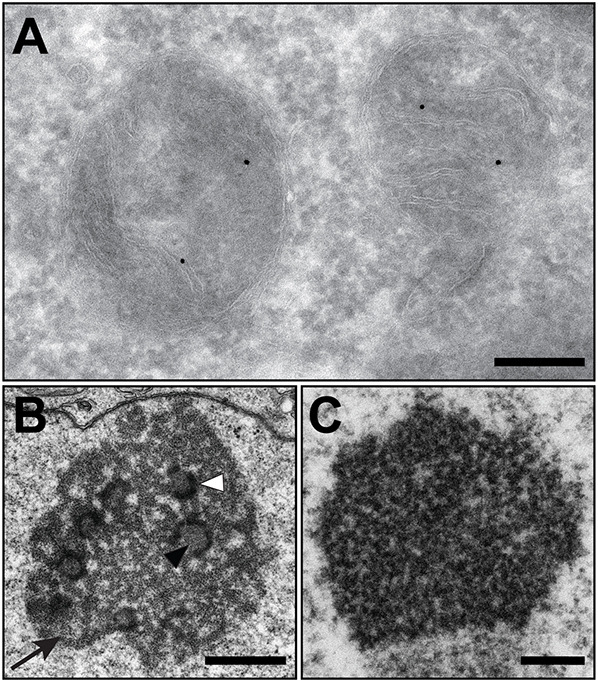
**Examples of the loss of reference space at high magnifications necessary for structural identification.** HeLa cell. (A) Tokuyasu technique immuno-EM, visualizing Cox4 on mitochondria with 10 nm gold particles. (B) Subcompartments of the nucleolus (from Fig. 2) as viewed by conventional TEM. Black arrowhead, fibrillar centre; white arrowhead, dense fibrillar component; black arrow, granular component. (C) Fine structure of the Cajal body as seen by conventional TEM. Scale bars: 200 nm (A,C); 1 µm (B).

The reference space of the structure of the interest is usually of enormous importance, which is particularly evident in immuno-EM, where the typical size of the gold particles used as reporters is in the range of 5–15 nm. For their detection, high magnifications are required so that only a small area of the cell and thus the reference space of the marker can be imaged simultaneously (Fig. 1A).

**Fig. 2.**
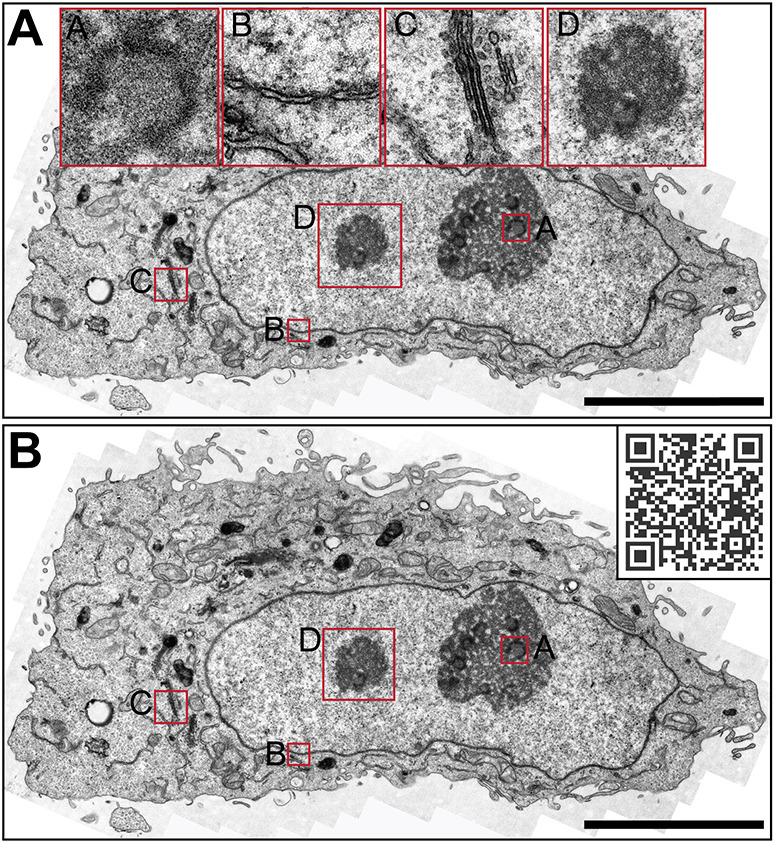
**Comparison of the traditional way of data presentation and dynamic browsing.** HeLa cell, as viewed by conventional TEM. (A) Representative figure with traditional inserts and annotations. (B) The same cell can be visualized with the EMMA method via https://wohlmann.github.io/2024_EMMA_F02 (also accessible through the included QR code). Scale bars: 5 µm (A,B, main image; insets A–C), 1 µm (inset D).

The same problem applies to ‘classical’ EM ultrastructure: for detailed structural information of organelles, high magnifications are required, but their position in the reference space or the distance to interaction partners can be important information, requiring overview data. One example of this issue is nuclear bodies, such as nucleoli and their subdomains, fibrillar centre, dense fibrillar component and granular component (Fig. 1B) or Cajal bodies, formerly called ‘coiled bodies’ for their appearance in EM (Fig. 1C). All of these domains were first described on the basis of their ultrastructure (Ghosh, 1976), and high magnifications are required to visualize these characteristic namesake features for their identification. However, as in immuno-EM, information about the reference space – the localization of the nuclear structures in relation to each other and within the nucleus – are important aspects related to their function (Bourgeois and Hubert, 1988; Janevski et al., 1997; Eskiw et al., 2003; Borsos and Torres-Padilla, 2016), information that is inaccessible at high magnifications, even though it is present in the sample.

In these and many other examples, different and distant structural aspects of a cell might be relevant to the same process; for example, in the intracellular localisation of a pathogen (Haas, 2009) or a nanoparticle (Griffiths et al., 2022), in the interaction of endocytic and biosynthetic compartments (Griffiths et al., 1989, 1993, 2022), in the formation and transport of virus particles in an infected cell (Griffiths et al., 2001b; Chlanda et al., 2009; Sodeik et al., 1993; Schmelz et al., 1994) or in the cellular effects of an inhibitor (Griffiths et al., 1983; Quinn et al., 1983). Such structural aspects might even interact directly with each other but might not be resolvable at a magnification allowing identification of their position relative to each other or to the whole cell; such relationships in the context of a tissue are of course even more problematic.

In general, the detailed view at higher magnifications and the sheer mass of structural information alone make it difficult to link the observed structural data to the bigger picture. A common metaphor for this problem is the ‘tree in the forest’ (Griffiths et al., 2001a).

In summary, to understand such complex relationships, information from different magnifications must be combined, ideally allowing a dynamic, continuous transition between them.

### Static solutions

The most common way in the field to visualize and describe a finding is to create panels and insets with high magnification images next to, or above a low magnification overview. However, with this type of visualization, relevant and important aspects might be missed and/or not reported because they are not detected at the magnifications used or simply overlooked during analysis. Furthermore, insets can obscure important aspects in the overview, which again can lead to difficulties in relating the overview to the details (Fig. 2A). To overcome this problem, an interactive, dynamic, continuous transition between magnifications and an interactive combination of overview and high-resolution data would be beneficial.

### Interactive solutions

The idea of an interactive solution to this issue(s) is not new and several approaches have been developed for light microscopy (LM), mostly for paraffin histology in either anatomy (Copper et al., 2018) or pathology (Gorman et al., 2023). For EM, this development was slightly delayed and unfortunately mostly based on commercial closed-source software as for the example of ‘nanotomy’, started in 2013 (Ravelli et al., 2013). With the increasing popularity of volume and 3D-EM, similar approaches are being introduced to conveniently analyse and browse the huge datasets (Vergara et al., 2021; Gupta et al., 2022; Salavert-Torres et al., 2016; Xu et al., 2021). Some more recent developments, such as the OME-Zarr file format (Moore et al., 2023) propose an even bigger step towards the noble aim of a universal open source file format compatible with multimodal data and big data exploration. However, all of these tools require a dedicated software that is often sophisticated and/or offers a wide range of options for filtering and editing, resulting in relatively complicated interfaces or processes for manipulating and visualizing the (image) data. Another powerful approach, in some cases supporting the mentioned OME-Zarr file format, are online hosting services for imaging data like BioImageArchive (Hartley et al., 2022), OpenOrganelle (Xu et al., 2021) or smaller OMERO (Burel et al., 2015) based approaches. These services typically offer additional functionality like the linking of multimodal datasets or 3D data browsing and markup strategies. Such services often require the data to be linked to a publication or at least to be of a certain quality and standard; data from earlier stages of a project might not fulfil these requirements but can still be very important for interactive discussions. Datasets that should not be publicly accessible, such as patient data or data from projects of commercial interest, can pose a further problem. Finally, online services usually require registration and carry the risk of being discontinued – especially if closed source formats are used for their visualisation, this can lead to problems and even loss of data. Here, we propose a simple and user-friendly approach to emulate the magnification steps between a low-magnification overview and high-resolution details, allowing dynamic browsing of the ultrastructure both locally (offline) and online. The solution is flexible in its adaptation and can be self-hosted (e.g. internally at an institution) allowing for full offline access for sensitive data, it does not require a single dedicated file format and is compatible with a wide range of commonly used file formats. Modifications and extensions of this simple approach allow flexible adaptation to more complex solutions by implementing a variety of functions as required. The fundamental concept is based on three operations and the corresponding tools.

The first step is the generation of gigapixel panoramas by image stitching, which is followed by sub-sampling and down-sampling to create readily viewable segments from the usually huge data set and finally a visualisation using a viewer plugin on a website (Fig. 2B). Such a website can then be used locally (offline or in an organisation's intranet), but it can also be hosted on any web server of the institution, a publisher or with any hosting or cloud service in order to be accessible via the internet.

## RESULTS

In order to visualize a large area at high magnification, several images must be stitched together to create one large image. The resulting image file is often huge and difficult to visualize and/or analyse for both technical and practical reasons. By creating virtual overviews with low magnification and dividing the individual file into many small areas, analysing and browsing the data set becomes much faster and more convenient. Additional functions and options that further facilitate the analysis can also be integrated into this visualization method. The three steps required to implement the visualization strategy are explained in detail below.

### Image stitching of EM data

Developments in image stitching for microscopy have focused mainly on fluorescence or light microscopy (histology), and the resulting approaches often fail when applied to EM data which are (by their very nature) often more complex, variable and precise. Moreover, the problem of inhomogeneous elastic deformations during imaging due to the interaction between the electron beam and the support film and/or sample (especially on slot grits, essential for imaging large areas) seems to cause problems when using stitching software. One exception is the well performing ImageJ or Fiji (Schindelin et al., 2012) software plugin BigStitcher (https://github.com/PreibischLab/BigStitcher; Hörl et al., 2019), which is able to reliably handle EM data. Obviously, the success of any application depends on the dataset in each individual case, for example, on the contrast, grey values, patterns and structures in the images.

**Fig. 3.**
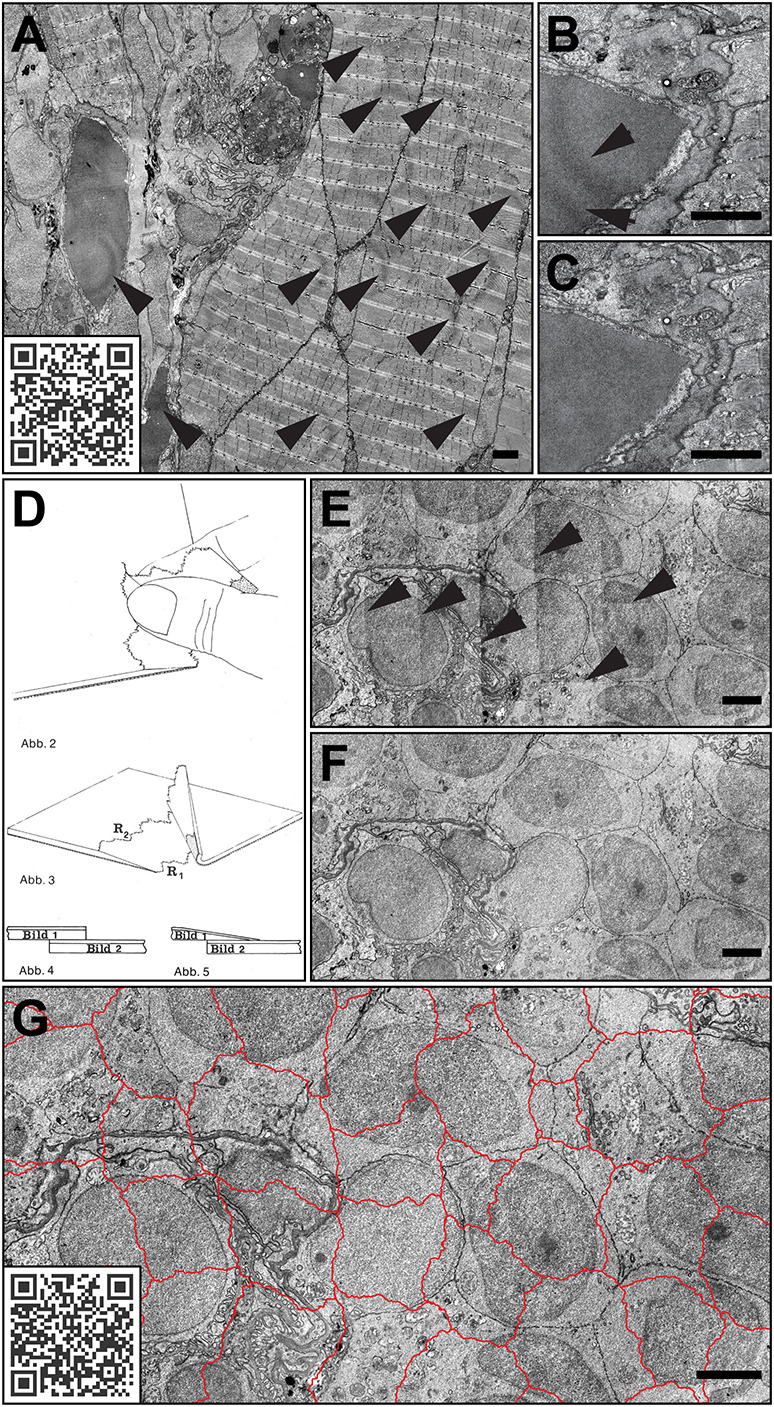
**Common projection artefacts and suggested solutions.** Tissue of a 5 days post fertilization (dpf) zebrafish larva, as viewed by conventional TEM. (A,B) Repetitive patterns due to inhomogeneous illumination exemplary indicated by arrowheads. (C) Image from B after rolling ball background subtraction. The image in A can be viewed with the EMMA method at https://wohlmann.github.io/2024_EMMA_F03A (also accessible through the included QR code). (D) Illustration of the tearing method, reprinted from Simonsberger et al. (Simonsberger et al., 1977) with kind permission from the authors. (E) Tile image projected with straight borders and without gradients; arrowheads indicate examples of undesirable steps in the brightness values at the edges of the merged images. (F) As in E but projected with irregular borders and gradients. (G) As in F, but with irregular borders used for projection are indicated in red. The EMMA method can be used for the comparison of E–G at https://wohlmann.github.io/2024_EMMA_F04 (also accessible through the included QR code). Scale bars: 2 µm.

There are some specialized software developments for stitching EM data, but these are mainly focused on the alignment and stitching of serial section reconstruction, tomography or volume data sets and can be complicated to use and inflexible (Saalfeld, 2019; Mahalingam et al., 2022; Vergara et al., 2021). Less often considered are stitching methods developed for photography, which are typically user-friendly and can also be applied to microscopy data (Gellrich, 2016; Legesse et al., 2015). Some of these software can handle EM images surprisingly well when the overlap between them is ∼30% and a rectilinear projection is used. Common issues in image stitching include the need for dealing with artificial repetitive patterns based on systematic inconsistencies in the image. These signals develop into repetitive patterns owing to the assembly process and are particularly pronounced in EM data. The most common source of this artefact is the inhomogeneous illumination of the primary images (‘illumination issue’) based on a misaligned beam or insufficient flat field correction of the detector – even if initially unnoticeable, such irregularities can become increasingly apparent during longer imaging sessions (Fig. 3A,B). Another common issue is the generation of artefacts at the stitching interfaces that result in lines or abrupt differences in brightness after projection of the resulting image (‘projection issue’) (Fig. 3D).

For the illumination issue, there are (besides the obvious alignment of the microscope and calibration of the detector) several well-functioning correction algorithms (e.g. via ImageJ), such as frequency domain filtering, polynomial shading correction or rolling ball background subtraction that can be applied to the primary images if necessary (Fig. 3C).

The projection issue, however, is still evident in many merged datasets today, and the seam lines are often clearly visible (Legesse et al., 2015). Unexpectedly, this problem was ingeniously solved long before the age of digital photography by creating irregular seam lines simply by physically tearing the images to be projected rather than cutting them (Simonsberger et al., 1977) (Fig. 3D). The resulting irregular lines will (in combination with a fading effect) conceal brightness differences and allow for smoother blending of the images during projection. The same principle can be applied in digital photo editing software such as GIMP (https://www.gimp.org/) or Photoshop (Adobe) and results in significantly improved projection quality (Fig. 3D–G).

### Dynamic visualisation of large image datasets

**Fig. 4.**
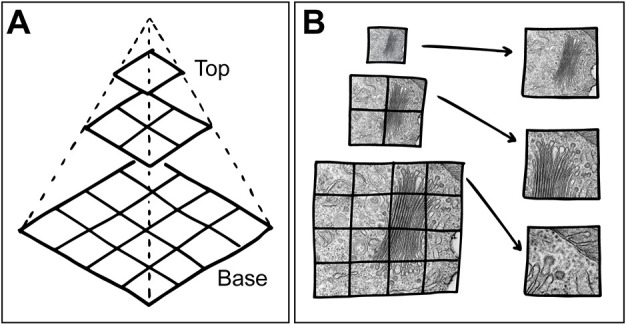
**The concept of image tile pyramids.** (A) Schematic illustrating the emulation of magnifications from a high-resolution base image plane (from bottom to top). (B) Schematic illustration of the process of loading image tiles from the respective layers into the viewer consequently simulating magnifications.

The resulting stitched (gigapixel-) image is a planar single file. Depending on the area imaged and the selected magnification, this file can be several dozen to hundreds of gigabytes in size. Such large files are difficult to analyse and require powerful hardware, as all data is loaded simultaneously when opened. To solve this problem and allow convenient exploration of the data, an image tile pyramid is generated from this file. Image pyramids are a tool that has been used extensively and for many purposes since the early days of image processing – there are a variety of approaches, most of which involve iteratively filtering of an original image to create a modified, e.g. smaller version (Adelson et al., 1984). Image tile pyramids are a combination of this approach (usually downscaling via a Gaussian pyramid) with tiling of the image into defined parts (tiles) after each downscaling iteration, resulting in multiple image mosaics on each layer corresponding to the virtually generated lower magnification (Fig. 4A). The position of the image tiles on the respective level and the link to the derived image tiles on the levels above takes place via a virtual coordinate system. At low magnification, only a few tiles with lower resolution (from a high pyramid level) are loaded in order to obtain an overview of the entire area; when zooming in, corresponding tiles with higher resolution are then loaded from the lower pyramid levels, whereby a higher magnification of the respective area is displayed (Fig. 4B). In this process, new coordinates overwrite the current positions, and the loaded tiles only ever correspond to at most a tiny part of the data set or a virtual representation with low magnification and resolution. This enables a fast and dynamic browsing of such large datasets.

A well-known application of such an approach is modern digital navigation tools like OpenStreetMap (https://www.openstreetmap.org/); similar to these navigation tools, microscopy ‘map’ data can also be visualized with JavaScript-based viewers on a simple website, hence we call this approach EMMA for ‘electron microscopy map’.

For this visualization strategy, a variety of software can be used (for an overview see Table S1). For stitching, as mentioned above, the ImageJ plugin BigStitcher (Hörl et al., 2019) is an excellent option, but the open-source software Hugin (https://hugin.sourceforge.io/) is also very suitable, whereas the easiest, most convenient solution is provided by the free Image Composite Editor software (https://www.microsoft.com/en-us/research/project/image-composite-editor/) from Microsoft Research.

The resulting modified image data sets can either be projected (‘blended’) directly in this software, or exported as a multilayer file (or a series of single-layer files) and then manually projected in the preferred image editing software (GIMP, Photoshop, etc.).

The subsequent generation of a multi-level image tile pyramid from the resulting flat gigapixel image can also be carried out using various software. Particularly worth mentioning is the ability of the Image Composite Editor, which could already be used for stitching, to export files directly in the open source dzi format. The dzi (Deep Zoom Image) format is an xml-based image tile pyramid system that can be displayed directly by most viewers. In addition, there are excellent open-source tools such as the Python package Deepzoom (https://github.com/openzoom/deepzoom.py) or very convenient but commercial solutions, such as Zoomify (https://www.zoomify.com) for this process. Although it is a commercial closed-source software, Zoomify is mentioned here for its simplicity and the possibility to also use it as a visualization interface in the next step. Other options, such as the use of ‘legacy image pyramids’ (LIP) for existing images without pyramid generation or less compatible file formats and their respective viewers, such as iiif, tms and zif, are not covered here.

### Interaction and visualization interface

Interactive exploration of this data should ideally not require dedicated software, and fortunately most browsers are readily capable of visualizing EMMA presentations via JavaScript/HTML5. Again, there is various software that can be used (see Table S1), but all viewers run as embedded scripts on a simple HTML and CSS web page (e.g. Fig. 2B, QRcode; Table S2). Although CSS is not essential, it allows the viewer to be scaled to different screen sizes, making EMMA presentations fully compatible with mobile devices.

As already indicated in the last section, the most convenient solution is probably to use the viewer included in the closed-source tool Zoomify in combination with an image tile pyramid created with either Zoomify or the Image Composite Editor. However, as we value the use of open-source software, our tool of choice is the excellent and user-friendly tool OpenSeadragon (https://openseadragon.github.io/), which is commonly used to visualise histology data. This script is not only open source, but also compatible with many tile pyramid formats, including those created with Zoomify or the Image Composite Editor (dzi format). However, it is not only because of the open-source aspect that we prefer OpenSeadragon; the simple implementation and the possibility of integrating various further features such as overlays, mark-ups, annotations and filters, make it an ideal solution in our opinion. There are other (open source) options that allow even more flexibility and customization, such as OpenLayers (https://openlayers.org/), which in our opinion is the ultimate solution when it comes to additional functionality and customization. However, the gain in versatility and functionality comes at the expense of simplicity, and since our goal is a simple, easy to implement and convenient solution, the more complex viewers will not be discussed here in detail. As both OpenLayers and OpenSeadragon are open-source software, there is also a wealth of custom plug-ins, extensive online documentation and a very active and helpful community with tutorials, advice and forums relating to these tools. One of these plug-ins is the useful plug-in OpenSeadragonScalebar (https://github.com/usnistgov/OpenSeadragonScalebar), which generates a floating, dynamic scalebar. It can be implemented with just a single script file and two lines of code (Table S2, row 1, html, lines 10+17–21) and is easily customized with just a few variables (Table S2, row 4, html, lines 61–65). The complexity of the script is not significantly increased by implementation of the scale bar, but the result is much more useful (Fig. 2, QRcode) than the static scale bar somewhere on the base image (Fig. 2, QRcode, bottom centre), therefore this is our default plugin in all EMMA presentations.

**Fig. 5.**
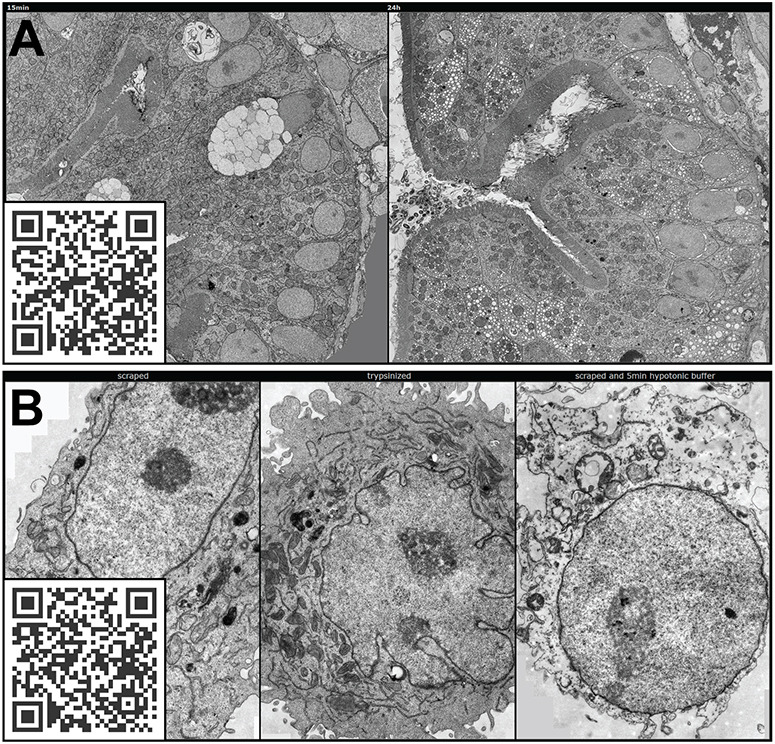
**Browser windows containing multiple viewer containers to allow direct comparative analysis.** (A) Two containers with the intestine of a 5 days post fertilization (dpf) zebrafish larva, showing a conventional TEM image at two time points. This can be visualized with EMMA method via https://wohlmann.github.io/2024_EMMA_F06B (also accessible through the included QR code). (B) Three containers with HeLa cells after different treatments (left, from Fig. 2) as viewed with conventional TEM. This can be visualized with EMMA method via https://wohlmann.github.io/2024_EMMA_F06C (also accessible through the included QR code). Scale bars can be seen when accessed via the provided URLs.

A common tool in biological research is the comparison between experimental conditions and time points or between treatment and control images. Such evaluation can be a challenging task, which is greatly facilitated by the use of EMMA presentations, when instead of a single container (embedded viewer), multiple containers are embedded on the same website. This visualization holds enormous potential, as primary microscopy data from different experimental conditions, time points or tissues can be interactively and dynamically correlated for exploratory analysis, greatly simplifying the direct comparison of data sets (Fig. 5).

**Fig. 6.**
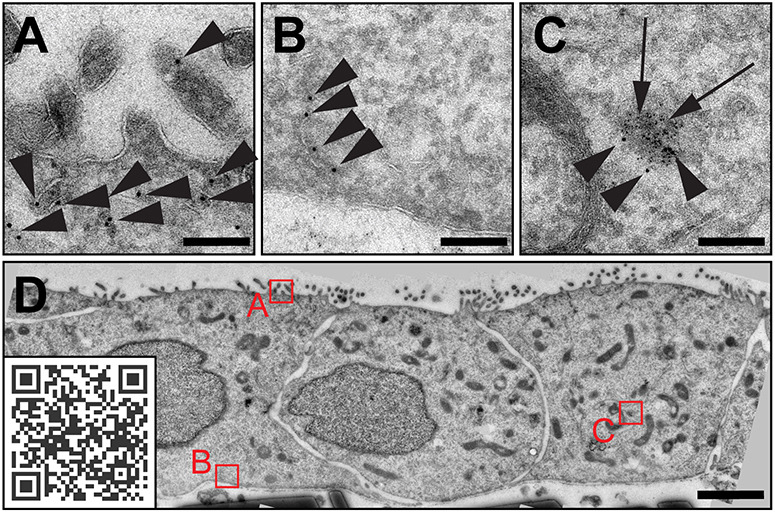
**Application of dynamic browsing to the most profiting methodology.** Tokuyasu technique immuno-EM, visualizing Rab11 on polarized endothelia cells (LLC-PK1) wih 10 nm gold particles (arrowheads) and late endocytic compartments with endocytosed 5 nm gold particles (arrows). (A) Intense labelling for Rab11 on membrane domains at the apical cell surface. (B) Labelling for Rab11 on membrane domains at the basolateral cell surface. (C) Labelling for Rab11 on endocytic membrane domains identified by 5 nm gold particles. (D) Overview. This can be visualized with EMMA method via https://wohlmann.github.io/2024_EMMA_F07D (also accessible through the included QR code). Scale bars: 500 nm (A–C), 2 µm (D).

**Fig. 7.**
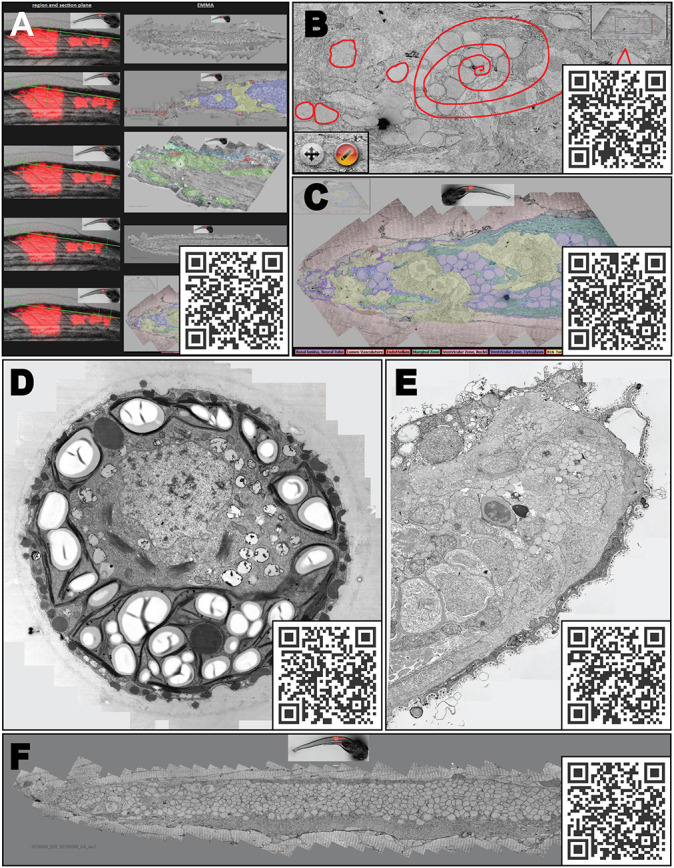
**Application to more complex scenarios and additional functionality for specific tasks.** (A) Example of an overview (web) page as a project overview, linking several maps and the respective position of the EM data in the light microscopy, green lines on the light microscopy data indicate sectioning plane and area, colour overlays to highlight structures of interest on the EM data, the EM images are links to the respective EMMA pages. This overview page as shown in the figure can visualized at https://wohlmann.github.io/2024_EMMA_F08A. (B) Interactive drawing plugin (‘openseadragon-annotations’) for overlays, controlled by two additional buttons (inset) to switch between drawing and dragging modes. QR-Code: EMMA-Method with interactive drawing plugin as shown in B. See https://wohlmann.github.io/2024_EMMA_F08B. (C) A more elaborate version allowing to toggle overlays on different regions of interest (code in Table S2, row 4). See the EMMA method presentation with switchable overlays at https://wohlmann.github.io/2024_EMMA_F08C. (D–F) Examples of the EMMA method with samples with increasing complexity. (D) Single-cell alga. See the EMMA method presentation at https://wohlmann.github.io/2024_EMMA_F09A. (E) Tissue region [fin of a 5 days post fertilization (dpf) zebrafish embryo]. See the EMMA method presentation at https://wohlmann.github.io/2024_EMMA_F09B. (F) Frontal section through the region of interest in a whole 5dpf zebrafish embryo bearing a xenograft tumour. See the EMMA method presentation at https://wohlmann.github.io/2024_EMMA_F09C. All URLs are accessible through the included QR codes. Scale bars can be seen when accessed via the provided URLs.

An essential aspect for all these approaches is the availability and communication of the respective EMMA presentations. For this purpose, it can be used either locally and offline or within a local intranet – due to their data security, such approaches are favourable for confidential datasets or patient data. To share the data with remote collaborators, the base map and viewer script need to be hosted online, for which there are many options – the simplest would again be a local server at the institution and a further simple and convenient solution is GitHub Pages (https://docs.github.com/en/pages; see Materials and Methods, point 10 for practical details). For either solution the URL can be distributed as a link or via a QR code on printed media or during talks (Figs 2–7). More conveniently, EMMA presentations can also be directly embedded in a website, such as in a figure of the online version of an article. If the dataset is expected to generate a massive amount of traffic or to combine advanced analyses and multimodal data, more advanced solutions such as the OME-Zarr format might be required, which in turn require a more advanced hosting and visualization method such as an S3 object storage, followed by visualization using tools, such as Neuroglancer (https://github.com/google/neuroglancer). As such advanced options are beyond the scope of this publication, they will not be discussed further.

## DISCUSSION

### Communicating and interpreting primary data

The direct, dynamic comparison of (ultra)structural data side-by-side and across a range of magnifications provides not only a convenient way for data exploration, but also a new way of communicating microscopy data, for instance in online publications. Instead of the usual presentation of a limited, often small, selected area, this enables the availability of extensive primary data, containing not only the reference space of the signal of interest, but ideally also multiple relevant events allowing the reader to browse and explore independently. With the above-mentioned implementation of multiple containers ‘side by side’, the comparison between different conditions is also greatly facilitated (Fig. 5). For example, when testing the effect of new drugs on cells and tissues at the ultrastructural level one is relying on recognizing patterns that might differ from a control sample. Such changes can be subtle and could be missed during the transition of magnifications or simply by the fact of not having the data directly comparable. Typically, some expected markers and housekeeping proteins are examined first using fluorescence microscopy, but unexpected phenotypes or structural changes are possibly overlooked with this approach (Pawellek et al., 2017). Although our proposed visualization strategy can technically be applied to fluorescence microscopy data by using stitching and projection software commonly used in light microscopy to create an image tile pyramid that is visualized with one of the scripts proposed here (Table S1), it is less useful for questions at the cellular level. This is primarily due to the almost binary signals, the lack of reference space and the lower resolution of fluorescence microscopy data (Griffiths, 2001). At lower magnification, such images are still more useful and easier to interpret than EM data (Griffiths et al., 1993). There are exceptions when analysing tissues or large areas that require high resolution; in such cases, our visualization method can be useful. Examples include the analysis of a cellular structure in adjacent tissues or in a whole organism, such as a zebrafish larva, simultaneously requiring low- and high-magnification information.

For electron micrographs, this situation is the norm. In classical transmission EM (TEM) analysis the upper limit of its resolution range is defined by the specimen carrier at 2.9 mm and the biologically relevant lower limit of a few nanometres is determined by the sample preparation rather than the microscope. Furthermore, the EM data itself is much more complex, as the entire biological structure and information is available rather than just selected signals. Although this is a tremendous advantage, it makes EM data more sensitive than fluorescence microscopy data and less useful when presented at lower resolution, as is evident in the small figures of most modern publications. Whereas EM photomicrographs were presented as several full-page images in the older cell biology literature (Clementi and Palade, 1969), they are now commonly squeezed into postage stamp-sized tiles of composite images, often rendering them impossible to interpret. Moreover, in most studies, the nature of the subject requires high-magnification images, for example of an endosome or a mitochondrion, which are commonly referred to as ‘representative images’. However, these images are often not really representative, but have been selected to best confirm the preferred hypothesis. In the rare cases where low magnification images are used to illustrate the structural context of the observed phenomenon, the opposite problem occurs – the fine structural details of, for example, membranes or cytoskeletal components become invisible or potentially important areas are obscured by high-magnification insets (Fig. 2A).

### Immuno-EM, the most powerful but most-affected method

An invaluable tool in microscopy is immuno-EM, the only method available to visualise a molecule of interest embedded in its reference space at ultrastructural resolution. This powerful method is the most affected by all the problems mentioned above, as the molecule of interest is labelled and visualized by a metal particle, often gold, in the size range of 5–15 nm, with a smaller particle allowing more precise localization and more efficient labelling (Slot and Geuze, 1981). To detect these small particles, magnifications of ∼10,000–15,000× are required, where 1 µm corresponds to 1–1.5 cm of the image and consequently only a tiny area of the reference space around the signals can be visualized. (Figs 1A, 5A–C). At such magnifications, a single cell appears like a complex tissue in light microscopy, with different compartments and regions interacting dynamically in various ways. For example, polarized epithelial cells, which have two independent membrane domains (the basolateral and the apical) (Farquhar and Palade, 1963), also have two separate endocytic pathways whose function is necessary to maintain the polarity of the cell (Cao et al., 2012). These pathways are controlled by the same cytoplasmic pool of factors, such as the Rab proteins. Thus, when studying these processes, the distribution of the molecules of interest within the cell is as important as their local concentration at (membrane) domains. The localization and identity of such endocytic membrane domains, which can be assessed by introducing metal particles of different sizes into the liquid phase of the endocytic system of the cell, also plays a crucial role.

Consequently, the information required to analyse the distribution of the target molecule is derived from different magnifications. A very high magnification is necessary to detect the small particles and analyse the fine structural details of their immediate environment, a medium magnification is needed to capture the labelled structure and a lower magnification is required to understand the localization of the labelled structures in relation to the whole cell.

One example is Rab11, which is thought to be involved in recycling from the early endosome and is localized in the apical region of a polarized cell (Fig. 6A). In the porcine epithelial cell line LLC-PK1, Rab11, which was labelled with 10 nm gold particles, is also found on basolateral membranes, albeit to a much lesser extent (Fig. 6B). It is also detected on late endocytic compartments identified with the fluid phase tracer 5 nm BSA–Gold, added to the cells as a marker for late endosomes and lysosomes (Fig. 6C). A figure containing all this information would be incredibly crowded and difficult to interpret. It would also only reflect the microscopists interpretation, possibly from a single cell and again face the ‘representative image problem’. Apart from the obvious need for appropriate sampling and quantification methods, e.g. by stereology (Griffiths et al., 2001a), it would be desirable to allow the reader or collaborator to look into the microscope themselves – this would allow the signal as well as the off-target labelling to be observed for many different events in several different cells (Fig. 6C, QRcode). A more detailed study of Rab11 is in preparation.

### Our proposed approach to solve these issues

The application of EMMA presentations will help to overcome the restrictions for all the situations mentioned above, as a large amount of image data, and therefore context, is communicated along with the figure, allowing independent browsing by the reader. Ideally, this viewer would be embedded directly in online publications, but it could also be implemented as a URL or more conveniently as a QR code in the image, for example in printed media (Figs 2–7). The accessibility of the primary data and the reference space not only facilitates the comprehension of interpretations, it also makes the data accessible for other microscopists, which might lead to additional conclusions and interpretations within the respective research project or even after publication.

Unfortunately, instead of efforts to improve direct communication and easy availability of primary data, a current trend is to use reconstructions or models of single individual events to convey an observation and illustrate general conclusions about a process. There are numerous tools available for segmenting EM data (Heinrich et al., 2021; Berger et al., 2018; Cardona et al., 2012; Mai and Kang, 2017) and significant efforts are being made to implement deep learning for this cumbersome task (Cheng et al., 2019; Pavarino et al., 2023; Spiers et al., 2021). The results are often fashionable and provide smoothed 3D renderings against a background of some primary data, which itself is often uninterpretable and sometimes of dubious quality. Although these approaches can be useful for certain questions, e.g. in the field of connectomics, to visualize a 3D structure using 2D information, they have a massive drawback that seems to be largely ignored – they analyse a single, isolated event rather than its entire population. This results in a similar problem to the infamous ‘representative image’ (which is biased by definition), but here an enormous amount of technology and labour is used to create such illustration. With a minute fraction of this effort, cheaper and more readily available instrumentation and less-demanding sample preparation, the simple application of stereology can provide an accurate quantitative morphometric analysis of the entire population of the event or structure (Griffiths et al., 2001a; Lucocq et al., 2015; Mayhew and Lucocq, 2015; Griffiths et al., 1984). These quantitative data can then be supplemented by corresponding primary data, e.g. by the approach proposed here, and ultimately lead to a more relevant, reliable and transparent result.

A drawback of our proposed method, common to all methods that use elastic alignment or segmentation, including in fluorescence microscopy, is the problem of quantification. Manipulation of the raw data during alignment, projection, reconstruction or segmentation, either by an algorithm or manual processes, makes it impossible to obtain quantitative data from these manipulated datasets. This problem is particularly pronounced when pixel-based software such as GIMP or Photoshop is used for microscopy data, but in our approach, it can be easily overcome by using the raw data for quantification (e.g. by stereology) before creating the EMMA presentation, which can then be utilized to illustrate the quantitative information.

Although there are open-source tools that allow similar browsing of EM data, of which the ImageJ plugin MoBIE (https://github.com/mobie/mobie-viewer-fiji; Vergara et al., 2021; Pape et al., 2023) is probably the best known and most powerful, the complexity of their implementation and use, the data type limitations and the relatively low (or complicated) customization capabilities for embedding in other media are hurdles to their regular use and implementation.

Obviously, the integration of further interactive features, tools and overlays is desirable and would make our proposed solution more versatile; such extensions, however, would be at the expense of simplicity and complicate the implementation (Table S2, row 4).

Our goal is to provide the simplest and easiest to use version possible, allowing anyone to implement a low-cost, low-technology solution for primary data communication and dynamic ultrastructure exploration.

This ‘basic’ implementation of EMMA presentations already allows for huge improvements, and apart from its potential in the context of publications (Kocere et al., 2020; Resseguier et al., 2023), it can also be a powerful tool to support interactive discussions (both on-site and online) and data exploration in research projects. For example, a page can be created that combines light microscopy data with electron microscopy overview data, where each EM overview image links to the corresponding EMMA presentations (Fig. 7A). Beyond that, it can also be particularly useful in teaching, as it best reflects the situation on the microscope and allows interactive magnification changes, free movement in the data set and comparison of different regions of interest of the same cell or tissue at different magnifications.

### Further possible developments for interactivity

There are many additional tools available for some of the open-source viewer plugins (e.g. OpenSeadragon or OpenLayers) that enhance the ‘on the microscope’ experience and/or provide a wealth of additional functionality. Their individual use is of obvious benefit, but as the possibilities are unlimited and fully customizable (see the documentation of the respective viewer), their implementation depends on the aims of the project. To illustrate the potential of some of the plugins, we have implemented the interactive drawing of overlays with ‘openseadragon-annotations’ (Fig. 7B), the overlays are temporarily saved and automatically deleted at the end of the session. Two buttons allow the user to switch between drawing and dragging mode (Fig. 7B, inset). This feature can be particularly useful in teaching or online discussions.

Overlays are generally a very helpful tool to highlight areas of interest, which in the form of coloured regions is a common way to annotate EM data in static images (Lerner et al., 2016; Kocere et al., 2020; Griffiths et al., 2022; Bhandari et al., 2023; Resseguier et al., 2023; Karreman et al., 2016) (Fig. 7A). Also, when combining light and electron microscopic data for correlative light and electron microscopy (CLEM), this approach is the most common. There are various ways to apply such manual overlays in EMMA presentations, for example using OpenLayers or one of the various OpenSeadragon plugins. However, even if transparent, these overlays can interfere with the unbiased interpretation of the primary image data, so it would be desirable to also see the image without overlays. By combining the overlay function of OpenSeadragon using image tile pyramids, generated from transparent overlay regions, with HTML buttons on a website, these overlays can be made switchable (Fig. 7C, QRcode). Such functionality can, for example, be realized by a JavaScript inline configuration on a website (Table S2, row 4).

The two examples are only a small glimpse of the extensive customization options that are available through various plugins. Although certainly helpful, they are fairly simple implementations of interactivity. An almost unlimited potential for mark-ups and annotations can be achieved by utilizing the extensive possibilities of HTML5 (the current standard markup language for websites) and its ‘canvas’ element through scripts, such as paper.js (https://github.com/paperjs/paper.js) or fabric.js (https://github.com/fabricjs/fabric.js/). However, these advanced features come at the price of increased complexity.

Much simpler plugins for OpenSeadragon and OpenLayers already offer a range of functionalities, including image filtering with ‘OpenSeadragonFiltering’ and spatial measurements through OpenLayers. As mentioned above, it is important to note that functions involving measurements should be used with caution, as the underlying images are manipulated datasets that are not suitable for accurate quantitative analysis. This is also the reason why stereological measurements, which could easily be deployed in a similar way, are not an application we are pursuing. Such quantitative data, however, can still be derived from the raw images, which are then visualized using EMMA presentations.

Ultimately, it can be imagined that this method of data exploration could also be implemented in the online version of publications, which would enable an immersive experience and exploration of the primary data while reading the respective publication.

### Scope and comparison to existing solutions

Although our proposed solution has strong potential for flexibility to specific tasks, it is intended to be a simple, easy to implement and low-technology tool. It aims to facilitate communication within projects by allowing dynamic browsing of primary data, making it an ideal aid for teaching purposes as well. Embedded visualization in publications, either directly in online versions or via QR codes in print media, posters and lectures, can be self-hosted which simplifies implementation. The visualization concept of utilizing image pyramids and OpenSeadragon, is already common in many fields such as geography, astronomy and histology, and could be easily adapted by journals to significantly enhance the publication of (EM) imaging data. This implementation would provide primary data for figures and could be further combined with tools like shinyHTM for R (https://github.com/embl-cba/shinyHTM; doi:10.5281/zenodo.2594651), to link quantitative data points to respective image data. Both approaches would improve the understanding and interpretation of the data after publication, in line with the ‘findable, accessible, interoperable, resuable’ (FAIR) principle (Wilkinson et al., 2016). A notable attempt project adhering to the FAIR principles is the OpenMicroscopyEnvironment community (Swedlow et al., 2003), which developed the OME-Zarr file format (Moore et al., 2023). This file format enables similar visualizations to our solution but aims for enabling such solutions in a universally compatible way by the sensible approach of complex metadata integration. The result is a powerful, open-source tool for multimodal datasets with almost unlimited potential. Although this is a very promising approach worth supporting, especially as it is fully open source, its advanced functionality, compatibility with multimodal data and advanced analyses lead to a more complex implementation and interface that can quickly become intimidating and complicated to understand. For simple 2D EM datasets, such advanced functionality is often unnecessary. Our proposed method is aiming at such datasets and is not intended to compete or compare to large-scale data repositories and generalized approaches. Rather, it is intended to be a simple tool supporting data communication within projects by offering an easy to implement and straightforward option that has extensive compatibility with existing workflows and file formats as well as a simple, intuitive interface. The possibility of local hosting and full offline data exploration is additionally beneficial for confidential datasets, such as patient data or data from projects of commercial interest, as well as for early-stage internal communication or data reviews. We hope that this basic concept will also be adapted for final datasets in publications in order to enhance multi-scale imaging data communication, which is especially valuable for EM data given its wide magnification range.

### Conclusion

Similar to a road map, where the small streets disappear in the mass of information when an entire city or even a country is to be visualized in its entirety, microscopy data also loses resolution at lower magnification. EM data is most affected by this problem due to its incredible magnification range and structural complexity. Inspired by digital map services, we attempted to solve this problem by implementing a similar visualization strategy enabling interactive, dynamic visualization of EM data. This simple and highly adaptable method is compatible with all image formats and easy to implement. It not only facilitates the analysis of EM data, but more importantly enables independent browsing of primary microscopy data where otherwise only few static ‘representative’ images are available. For example, if a publication claims that an organelle has changed as a result of a certain treatment, usually only a single high-resolution micrograph is available to assess the quality of the data. By contrast, the proposed method allows the reader to dynamically view an extended area showing several such organelles and their reference space at different magnifications. This experience resembles observing a sample under a microscope and allows researchers to follow the interpretations or even draw further conclusions, which makes it an ideal tool for data communication and also for interactive discussions and teaching. However, the most important aspect is that the primary data and the reference space become accessible to other microscopists, possibly even from unrelated research areas, either in the context of the respective research project with collaborators or even after publication. Applying this visualization method, for example in online versions of publications, is technically straightforward and would enable interactive reading, making interpretations and conclusions more transparent. In printed media or on posters and slides, visualization can take place via a URL or, even more conveniently, via a QR code (Figs 2–7).

The applications and possible uses of the data visualization method described here are merely the tip of the iceberg. With increasing complexity of the sample, the advantages of this method and its flexible customization options will gain importance. By extending the field of view from single-celled organisms (Fig. 7D) to tissues (Fig. 7E), organoids and even parts of (Fig. 7F) or entire model organisms, a significantly improved visualization and communication of ultrastructural data can be achieved (Kocere et al., 2020; Resseguier et al., 2023). Developments in automated acquisition enable effortless imaging of large areas, even in EM, and the possibility of, for example, screening an entire tumour and its microenvironment or even an entire zebrafish embryo at the ultrastructural level is not unrealistic, which we believe holds enormous potential. Of course, the logarithmically increasing size of the image datasets represents a processing obstacle for this visualization. However, the resulting datasets are comparatively small and the hardware requirements for processing correspond to the average requirements in most imaging laboratories. We believe that the technical effort is justifiable as it enables the exploration of primary data. This is in contrast to most modern EM-based visualizations, which usually require the same or even better hardware, but generate individual models of isolated structures instead of visualizing a population of these structures embedded in their reference space.

## MATERIALS AND METHODS

### A short practical overview

There is a variety of different approaches and software that can be used, a non-exhaustive overview of our preferred software can be found as an overview in Table S1, with URLs in Table S3, with the simplest solution printed in bold and optional steps in italics. This overview does not cover the plethora of advanced scripts for the respective tasks, but contains both very simple as well as advanced, customizable tools. For practical application, all necessary information can be found in the documentation of the respective tool.

Below, however, we propose a minimal step-by-step recipe for a free and open-source version with a floating scale bar. Furthermore, in the supplement, we provide minimal HTML examples for websites with a dzi and OpenSeadragon-based container with floating scale bar, a Zoomify-based container and two Zoomify-based containers (Table S2). These examples, as well as alternatives and extended versions for multiple viewers or additional functions, are also available for download under https://github.com/wohlmann/2024_EMMA_BOXES. In all examples, the italic text should be adapted to the respective project and the red parameters to the individual requirements. The examples use CSS styles to allow scaling to different screen sizes and make EMMA presentations that are fully mobile device compatible.

### A minimal step-by-step recipe for a free and open-source version with floating scale bar

1. record a tile dataset with about 30% overlap (automatic or manually)
2. download software:
  2.1 Microsoft ICE msi (https://archive.org/download/ice-2.0.3-for-64-bit-windows)
         2.1.1 Install the software
  2.2 OpenSeadragon.zip (https://openseadragon.github.io/#download)
  2.3 openseadragon-scalebar.js (https://github.com/usnistgov/OpenSeadragonScalebar)
3. Generate following folder structure (*NAME* of your choice):
  …/*NAME*/dzi/
  …/*NAME*/openseadragon/
4. Copy the scripts:
  4.1 Copy openseadragon-scalebar.js in the openseadragon folder resulting in: …/*NAME*/openseadragon/openseadragon-scalebar.js
  4.2 Copy the content of the openseadragon.zip in the openseadragon folder resulting in:
  …/*NAME*/openseadragon/images/……
  …/*NAME*/openseadragon/ openseadragon.js
  …/*NAME*/openseadragon/ openseadragon.min.js
5. Generate a new file called index.html in …/*NAME*/ resulting in:
  …/*NAME*/index.html
  5.1 Open the file index.html in any text editor (e.g. notebook++ [https://notepad-plus-plus.org/downloads/])
  5.2 Copy the content of the desired example from Table S2 of this article in the index.html and save
6. Open Microsoft ICE and follow the interface to import, stitch, crop and export (export using the Deep Zoom option), the filename (without the .dzi) will later be used as ID
  Copy the just generated dzi file in the folder …/*NAME*/dzi/
7. Adjust the values in your index.html file (by using the text editor):
  7.1 The “90%” values (the size of the viewer on the page) can be kept or adapted as needed
  7.2 As the generated folder structure is identical to the code in the selected example in Table S2 also the URLs (*openseadragon/*, openseadragon/images/ and *dzi/*) can be kept
  7.3 Replace YOURID with the filename of your dzi file (without the .dzi)
  7.4 Set the PixelsPerMeter value depending your dataset (can be read out in e.g. ImageJ or by measuring the scalebar in pixels), use integer
  7.5 The offset values can be kept or adjusted like desired
  7.6 save the index.html file
8. open the index.html file in any browser
9. browse your dataset
10. *optionally: upload to a hoster and deploy from main for online access, as example GitHub is used here but the process is very similar for all hosting, a detailed instruction can be found at* https://pages.github.com/
  10.1 generate a free GitHub account
  10.2 create a public repository in GitHub
  10.3 upload the data folder, the opensedragon folder and index.html file to this repository
  10.4 in the settings of the new repository find the “Pages” category
  10.5 under “Branch” change from “None” to “main” in the dropdown
  10.6 visit https://USERNAME.github.io/REPOSITORYNAME/

### Specimen preparation for TEM

Cell lines used for EM were LLC-PK1 (kindly provided by Kristian Prydz, UIO, Oslo) and HeLa Kyoto (kindly provided by Angus Lamond, University of Dundee, UK). The zebrafish embryos are wild-type larvae (kindly provided by Federico Fenaroli, University of Stavanger, Norway). All animal experiments were performed according to approved guidelines.

For convention TEM, samples were fixed for 48 h with 1% glutaraldehyde and 4% formaldehyde in PHEM buffer, pH 7.4, quenched using 100 mM glycine in 50 mM HEPES buffer pH 7.2 and embedded in 1% low-melting point agarose in water. Postfixation was performed using 1% OsO_4_ and 1.5% potassiumferricyanide in water for 2 h on ice, followed by four washes with water and 2% uranyl acetate for 1 h at room temperature. After dehydration through an ascending alcohol series, the samples are infiltrated gradually over 2 days with EPON resin, placed in moulds and heat-polymerised at 60°C for 16 h. Then, 50 nm sections were mounted on pioloform and carbon coated copper grids, contrasted using 0.1% lead citrate for 15 s before imaging with a Jeol JEM-1400 at 120 kV using a Tvips 216 camera.

Tokuyasu method EM was performed as per Tokuyasu (1973).

## Supplementary Material



10.1242/joces.262198_sup1 Supplementary information

## References

[JCS262198C1] Adelson, E., Anderson, C., Bergen, J., Burt, P. and Ogden, J. (1984). Pyramid Methods in Image Processing. *RCA Engineer.* 29, 33-41.

[JCS262198C2] Berger, D. R., Seung, H. S. and Lichtman, J. W. (2018). VAST (Volume Annotation and Segmentation Tool): efficient manual and semi-automatic labeling of large 3D image stacks. *Front. Neural Circuits* 12, 88. 10.3389/fncir.2018.00088 30386216 PMC6198149

[JCS262198C3] Bhandari, M., Soria-Carrera, H., Wohlmann, J., Dal, N. K., De La Fuente, J. M., Martin-Rapun, R., Griffiths, G. and Fenaroli, F. (2023). Subcellular localization and therapeutic efficacy of polymeric micellar nanoparticles encapsulating bedaquiline for tuberculosis treatment in zebrafish. *Biomater Sci*. 11, 2103-2114. 10.1039/d2bm01835g 36723226

[JCS262198C4] Borsos, M. and Torres-Padilla, M. E. (2016). Building up the nucleus: nuclear organization in the establishment of totipotency and pluripotency during mammalian development. *Genes Dev.* 30, 611-621. 10.1101/gad.273805.115 26980186 PMC4803048

[JCS262198C6] Bourgeois, C. A. and Hubert, J. (1988). Spatial relationship between the nucleolus and the nuclear envelope: structural aspects and functional significance. In *International Review of Cytology* (ed. G. H. Bourne, K. W. Jeon and M. Friedlander), pp. 1-52. Academic Press. 10.1016/s0074-7696(08)61730-1 3074957

[JCS262198C7] Burel, J. M., Besson, S., Blackburn, C., Carroll, M., Ferguson, R. K., Flynn, H., Gillen, K., Leigh, R., Li, S., Lindner, D. et al. (2015). Publishing and sharing multi-dimensional image data with OMERO. *Mamm. Genome* 26, 441-447. 10.1007/s00335-015-9587-6 26223880 PMC4602067

[JCS262198C8] Cao, X., Surma, M. A. and Simons, K. (2012). Polarized sorting and trafficking in epithelial cells. *Cell Res.* 22, 793-805. 10.1038/cr.2012.64 22525333 PMC3343658

[JCS262198C9] Cardona, A., Saalfeld, S., Schindelin, J., Arganda-Carreras, I., Preibisch, S., Longair, M., Tomancak, P., Hartenstein, V. and Douglas, R. J. (2012). TrakEM2 software for neural circuit reconstruction. *PLoS One* 7, e38011. 10.1371/journal.pone.0038011 22723842 PMC3378562

[JCS262198C10] Cheng, H. C., Cardone, A., Jain, S., Krokos, E., Narayan, K., Subramaniam, S. and Varshney, A. (2019). Deep-learning-assisted volume visualization. *IEEE Trans. Vis. Comput. Graph* 25, 1378-1391. 10.1109/TVCG.2018.2796085 29994182 PMC8369530

[JCS262198C11] Chlanda, P., Carbajal, M. A., Cyrklaff, M., Griffiths, G. and Krijnse-Locker, J. (2009). Membrane rupture generates single open membrane sheets during vaccinia virus assembly. *Cell Host Microbe* 6, 81-90. 10.1016/j.chom.2009.05.021 19616767

[JCS262198C12] Clementi, F. and Palade, G. E. (1969). Intestinal capillaries. I. Permeability to peroxidase and ferritin. *J. Cell Biol.* 41, 33-58. 10.1083/jcb.41.1.33 5775791 PMC2107738

[JCS262198C13] Copper, J. E., Budgeon, L. R., Foutz, C. A., Van Rossum, D. B., Vanselow, D. J., Hubley, M. J., Clark, D. P., Mandrell, D. T. and Cheng, K. C. (2018). Comparative analysis of fixation and embedding techniques for optimized histological preparation of zebrafish. *Comp. Biochem. Physiol. C Toxicol. Pharmacol.* 208, 38-46. 10.1016/j.cbpc.2017.11.003 29157956 PMC5936644

[JCS262198C14] Eskiw, C. H., Dellaire, G., Mymryk, J. S. and Bazett-Jones, D. P. (2003). Size, position and dynamic behavior of PML nuclear bodies following cell stress as a paradigm for supramolecular trafficking and assembly. *J. Cell Sci.* 116, 4455-4466. 10.1242/jcs.00758 13130097

[JCS262198C15] Farquhar, M. G. and Palade, G. E. (1963). Junctional complexes in various epithelia. *J. Cell Biol.* 17, 375-412. 10.1083/jcb.17.2.375 13944428 PMC2106201

[JCS262198C16] Gellrich, M. M. (2016). A simple method for panretinal imaging with the slit lamp. *Int. Ophthalmol.* 36, 775-780. 10.1007/s10792-016-0193-8 26879088

[JCS262198C17] Ghosh, S. (1976). The nucleolar structure. *Int. Rev. Cytol.* 44, 1-28. 10.1016/S0074-7696(08)61645-9 770368

[JCS262198C18] Gorman, C., Punzo, D., Octaviano, I., Pieper, S., Longabaugh, W. J. R., Clunie, D. A., Kikinis, R., Fedorov, A. Y. and Herrmann, M. D. (2023). Interoperable slide microscopy viewer and annotation tool for imaging data science and computational pathology. *Nat. Commun.* 14, 1572. 10.1038/s41467-023-37224-2 36949078 PMC10033920

[JCS262198C19] Griffiths, G. (2001). Bringing electron microscopy back into focus for cell biology. *Trends Cell Biol.* 11, 153-154. 10.1016/S0962-8924(01)01949-3 11354032

[JCS262198C20] Griffiths, G., Quinn, P. and Warren, G. (1983). Dissection of the Golgi complex. I. Monensin inhibits the transport of viral membrane proteins from medial to trans Golgi cisternae in baby hamster kidney cells infected with Semliki Forest virus. *J. Cell Biol.* 96, 835-850. 10.1083/jcb.96.3.835 6682112 PMC2112386

[JCS262198C21] Griffiths, G., Warren, G., Quinn, P., Mathieu-Costello, O. and Hoppeler, H. (1984). Density of newly synthesized plasma membrane proteins in intracellular membranes. I. Stereological studies. *J. Cell Biol.* 98, 2133-2141. 10.1083/jcb.98.6.2133 6563037 PMC2113041

[JCS262198C22] Griffiths, G., Back, R. and Marsh, M. (1989). A quantitative analysis of the endocytic pathway in baby hamster kidney cells. *J. Cell Biol.* 109, 2703-2720. 10.1083/jcb.109.6.2703 2592402 PMC2115901

[JCS262198C23] Griffiths, G., Parton, R. G., Lucocq, J., Van Deurs, B., Brown, D., Slot, J. W. and Geuze, H. J. (1993). The immunofluorescent era of membrane traffic. *Trends Cell Biol.* 3, 214-219. 10.1016/0962-8924(93)90114-G 14731755

[JCS262198C24] Griffiths, G., Lucocq, J. M. and Mayhew, T. M. (2001a). Electron microscopy applications for quantitative cellular microbiology. *Cell. Microbiol.* 3, 659-668. 10.1046/j.1462-5822.2001.00142.x 11580751

[JCS262198C25] Griffiths, G., Roos, N., Schleich, S. and Locker, J. K. (2001b). Structure and assembly of intracellular mature vaccinia virus: thin-section analyses. *J. Virol.* 75, 11056-11070. 10.1128/JVI.75.22.11056-11070.2001 11602745 PMC114685

[JCS262198C26] Griffiths, G., Gruenberg, J., Marsh, M., Wohlmann, J., Jones, A. T. and Parton, R. G. (2022). Nanoparticle entry into cells; the cell biology weak link. *Adv. Drug Deliv. Rev.* 188, 114403. 10.1016/j.addr.2022.114403 35777667

[JCS262198C27] Gupta, Y., Costa, C., Pinho, E., Silva, L. A. and Heintzmann, R. (2022). IMAGE-IN: Interactive web-based multidimensional 3D visualizer for multi-modal microscopy images. *PLoS One* 17, e0279825. 36584152 10.1371/journal.pone.0279825 PMC9803232

[JCS262198C28] Haas, A. (2009). Everybody has a Home of Their Own – ‘The Phagosome Zoo’. In *Intracellular Niches of Microbes* (eds Schaible, U. E., Haas, A.), pp. 159-190. Wiley. 10.1002/9783527629176.ch10

[JCS262198C29] Hartley, M., Kleywegt, G. J., Patwardhan, A., Sarkans, U., Swedlow, J. R. and Brazma, A. (2022). The bioimage archive - building a home for life-sciences microscopy data. *J. Mol. Biol.* 434, 167505. 10.1016/j.jmb.2022.167505 35189131

[JCS262198C30] Heinrich, L., Bennett, D., Ackerman, D., Park, W., Bogovic, J., Eckstein, N., Petruncio, A., Clements, J., Pang, S., Xu, C. S. et al. (2021). Whole-cell organelle segmentation in volume electron microscopy. *Nature* 599, 141-146. 10.1038/s41586-021-03977-3 34616042

[JCS262198C31] Hörl, D., Rojas Rusak, F., Preusser, F., Tillberg, P., Randel, N., Chhetri, R. K., Cardona, A., Keller, P. J., Harz, H., Leonhardt, H. et al. (2019). BigStitcher: reconstructing high-resolution image datasets of cleared and expanded samples. *Nat. Methods* 16, 870-874. 10.1038/s41592-019-0501-0 31384047

[JCS262198C32] Janevski, J., Park, P. C. and Boni, U. D. (1997). Changes in morphology and spatial position of coiled bodies during NGF-induced neuronal differentiation of PC12 cells. *J. Histochem. Cytochem.* 45, 1523-1531. 10.1177/002215549704501109 9358854

[JCS262198C33] Karreman, M. A., Mercier, L., Schieber, N. L., Solecki, G., Allio, G., Winkler, F., Ruthensteiner, B., Goetz, J. G. and Schwab, Y. (2016). Fast and precise targeting of single tumor cells in vivo by multimodal correlative microscopy. *J. Cell Sci.* 129, 444-456. 26659665 10.1242/jcs.181842 PMC4732291

[JCS262198C34] Kocere, A., Resseguier, J., Wohlmann, J., Skjeldal, F. M., Khan, S., Speth, M., Dal, N. K., Ng, M. Y. W., Alonso-Rodriguez, N., Scarpa, E. et al. (2020). Real-time imaging of polymersome nanoparticles in zebrafish embryos engrafted with melanoma cancer cells: localization, toxicity and treatment analysis. *EBioMedicine* 58, 102902. 10.1016/j.ebiom.2020.102902 32707448 PMC7381511

[JCS262198C35] Legesse, F. B., Chernavskaia, O., Heuke, S., Bocklitz, T., Meyer, T., Popp, J. and Heintzmann, R. (2015). Seamless stitching of tile scan microscope images. *J. Microsc.* 258, 223-232. 10.1111/jmi.12236 25787148

[JCS262198C36] Lerner, T. R., De Souza Carvalho-Wodarz, C., Repnik, U., Russell, M. R., Borel, S., Diedrich, C. R., Rohde, M., Wainwright, H., Collinson, L. M., Wilkinson, R. J. et al. (2016). Lymphatic endothelial cells are a replicative niche for Mycobacterium tuberculosis. *J. Clin. Invest.* 126, 1093-1108. 10.1172/JCI83379 26901813 PMC4767353

[JCS262198C37] Lucocq, J. M., Mayhew, T. M., Schwab, Y., Steyer, A. M. and Hacker, C. (2015). Systems biology in 3D space--enter the morphome. *Trends Cell Biol.* 25, 59-64. 10.1016/j.tcb.2014.09.008 25455351

[JCS262198C38] Mahalingam, G., Torres, R., Kapner, D., Trautman, E. T., Fliss, T., Seshamani, S., Perlman, E., Young, R., Kinn, S., Buchanan, J. et al. (2022). A scalable and modular automated pipeline for stitching of large electron microscopy datasets. *Elife* 11, e76534. 10.7554/eLife.76534 35880860 PMC9427110

[JCS262198C39] Mai, K. K. K. and Kang, B. H. (2017). Semiautomatic segmentation of plant golgi stacks in electron tomograms using 3dmod. *Methods Mol. Biol.* 1662, 97-104. 10.1007/978-1-4939-7262-3_8 28861820

[JCS262198C40] Mayhew, T. M. and Lucocq, J. M. (2015). From gross anatomy to the nanomorphome: stereological tools provide a paradigm for advancing research in quantitative morphomics. *J. Anat.* 226, 309-321. 10.1111/joa.12287 25753334 PMC4386931

[JCS262198C41] Moore, J., Basurto-Lozada, D., Besson, S., Bogovic, J., Bragantini, J., Brown, E. M., Burel, J.-M., Casas Moreno, X., De Medeiros, G., Diel, E. E. et al. (2023). OME-Zarr: a cloud-optimized bioimaging file format with international community support. *Histochem. Cell Biol.* 160, 223-251. 10.1007/s00418-023-02209-1 37428210 PMC10492740

[JCS262198C42] Pape, C., Meechan, K., Moreva, E., Schorb, M., Chiaruttini, N., Zinchenko, V., Martinez Vergara, H., Mizzon, G., Moore, J., Arendt, D. et al. (2023). MoBIE: a Fiji plugin for sharing and exploration of multi-modal cloud-hosted big image data. *Nat. Methods* 20, 475-476. 10.1038/s41592-023-01776-4 36765247

[JCS262198C43] Pavarino, E. C., Yang, E., Dhanyasi, N., Wang, M. D., Bidel, F., Lu, X., Yang, F., Francisco Park, C., Bangalore Renuka, M., Drescher, B. et al. (2023). mEMbrain: an interactive deep learning MATLAB tool for connectomic segmentation on commodity desktops. *Front. Neural Circuits* 17, 952921. 10.3389/fncir.2023.952921 37396399 PMC10309043

[JCS262198C44] Pawellek, A., Ryder, U., Tammsalu, T., King, L. J., Kreinin, H., Ly, T., Hay, R. T., Hartley, R. C. and Lamond, A. I. (2017). Characterisation of the biflavonoid hinokiflavone as a pre-mRNA splicing modulator that inhibits SENP. *Elife* 6, e27402. 10.7554/eLife.27402 28884683 PMC5619949

[JCS262198C45] Quinn, P., Griffiths, G. and Warren, G. (1983). Dissection of the Golgi complex. II. Density separation of specific Golgi functions in virally infected cells treated with monensin. *J. Cell Biol.* 96, 851-856. 10.1083/jcb.96.3.851 6403555 PMC2112410

[JCS262198C46] Ravelli, R. B., Kalicharan, R. D., Avramut, M. C., Sjollema, K. A., Pronk, J. W., Dijk, F., Koster, A. J., Visser, J. T., Faas, F. G. and Giepmans, B. N. (2013). Destruction of tissue, cells and organelles in type 1 diabetic rats presented at macromolecular resolution. *Sci. Rep.* 3, 1804. 10.1038/srep01804 23652855 PMC3647201

[JCS262198C47] Resseguier, J., Nguyen-Chi, M., Wohlmann, J., Rigaudeau, D., Salinas, I., Oehlers, S. H., Wiegertjes, G. F., Johansen, F. E., Qiao, S. W., Koppang, E. O. et al. (2023). Identification of a pharyngeal mucosal lymphoid organ in zebrafish and other teleosts: Tonsils in fish? *Sci. Adv.* 9, eadj0101. 10.1126/sciadv.adj0101 37910624 PMC10619939

[JCS262198C48] Saalfeld, S. (2019). Computational methods for stitching, alignment, and artifact correction of serial section data. *Methods Cell Biol.* 152, 261-276. 10.1016/bs.mcb.2019.04.007 31326024

[JCS262198C49] Salavert-Torres, J., Iudin, A., Lagerstedt, I., Sanz-García, E., Kleywegt, G. J. and Patwardhan, A. (2016). Web-based volume slicer for 3D electron-microscopy data from EMDB. *J. Struct. Biol.* 194, 164-170. 10.1016/j.jsb.2016.02.012 26876163 PMC4819904

[JCS262198C50] Schindelin, J., Arganda-Carreras, I., Frise, E., Kaynig, V., Longair, M., Pietzsch, T., Preibisch, S., Rueden, C., Saalfeld, S., Schmid, B. et al. (2012). Fiji: an open-source platform for biological-image analysis. *Nat. Methods* 9, 676-682. 10.1038/nmeth.2019 22743772 PMC3855844

[JCS262198C51] Schmelz, M., Sodeik, B., Ericsson, M., Wolffe, E. J., Shida, H., Hiller, G. and Griffiths, G. (1994). Assembly of vaccinia virus: the second wrapping cisterna is derived from the trans Golgi network. *J. Virol.* 68, 130-147. 10.1128/jvi.68.1.130-147.1994 8254722 PMC236272

[JCS262198C52] Simonsberger, P., Lametschwandtner, A., Sulzer, G., Albrecht, U. and Adam, H. (1977). A simple method of tearing micrographs in order to produce large dimensioned photomontages of light and electron microscopical pictures. *J. Microsc. Res. Meth.* 33, 278-280. 414154

[JCS262198C53] Slot, J. W. and Geuze, H. J. (1981). Sizing of protein A-colloidal gold probes for immunoelectron microscopy. *J. Cell Biol.* 90, 533-536. 10.1083/jcb.90.2.533 7026575 PMC2111877

[JCS262198C54] Sodeik, B., Doms, R. W., Ericsson, M., Hiller, G., Machamer, C. E., Van ‘T Hof, W., Van Meer, G., Moss, B. and Griffiths, G. (1993). Assembly of vaccinia virus: role of the intermediate compartment between the endoplasmic reticulum and the Golgi stacks. *J. Cell Biol.* 121, 521-541. 10.1083/jcb.121.3.521 8486734 PMC2119557

[JCS262198C55] Spiers, H., Songhurst, H., Nightingale, L., De Folter, J., Hutchings, R., Peddie, C. J., Weston, A., Strange, A., Hindmarsh, S., Lintott, C. et al. (2021). Deep learning for automatic segmentation of the nuclear envelope in electron microscopy data, trained with volunteer segmentations. *Traffic* 22, 240-253. 10.1111/tra.12789 33914396

[JCS262198C56] Swedlow, J. R., Goldberg, I., Brauner, E. and Sorger, P. K. (2003). Informatics and quantitative analysis in biological imaging. *Science* 300, 100-102. 10.1126/science.1082602 12677061 PMC3522889

[JCS262198C60] Tokuyasu, K. T. (1973). A technique for ultracryotomy of cell suspensions and tissues. *J. Cell Biol.* 57, 551-565. 10.1083/jcb.57.2.551 4121290 PMC2108989

[JCS262198C57] Vergara, H. M., Pape, C., Meechan, K. I., Zinchenko, V., Genoud, C., Wanner, A. A., Mutemi, K. N., Titze, B., Templin, R. M., Bertucci, P. Y. et al. (2021). Whole-body integration of gene expression and single-cell morphology. *Cell* 184, 4819-4837.e22. 10.1016/j.cell.2021.07.017 34380046 PMC8445025

[JCS262198C58] Wilkinson, M. D., Dumontier, M., Aalbersberg, I. J., Appleton, G., Axton, M., Baak, A., Blomberg, N., Boiten, J. W., Da Silva Santos, L. B., Bourne, P. E. et al. (2016). The FAIR Guiding Principles for scientific data management and stewardship. *Sci. Data* 3, 160018. 10.1038/sdata.2016.18 26978244 PMC4792175

[JCS262198C59] Xu, C. S., Pang, S., Shtengel, G., Müller, A., Ritter, A. T., Hoffman, H. K., Takemura, S.-Y., Lu, Z., Pasolli, H. A., Iyer, N. et al. (2021). An open-access volume electron microscopy atlas of whole cells and tissues. *Nature* 599, 147-151. 10.1038/s41586-021-03992-4 34616045 PMC9004664

