# Supplementary material for: Expanding the field of view – a simple approach for interactive visualisation of electron microscopy data
Source: J Cell Sci. 2024 Oct 23;137(20):jcs262198. doi: 10.1242/jcs.262198 (PMC11529876; doi:10.1242/jcs.262198)
Supplement: Supplementary information [file joces-137-262198-s1.pdf]

Table S1. Overview of a selection of useable software tools, the **simplest** solution is printed in bold and *optional* steps in italics

Step	Software
Record	- Microscope manufacturer<br>- SerialEM
Align	- Microscope manufacturer<br>- Big Stitcher<br>- Hugin<br>- **Image Composite Editor**<br>- SerialEM
Correct if necessary (e.g. in ImageJ)	- *polynomial shading correction*<br>- *Rolling ball background subtraction*<br>- *Fourier filtering*
Stitch / Project	- Microscope manufacturer<br>- Big Stitcher<br>- Hugin<br>- Panotools<br>- GIMP<br>- **Image Composite Editor**<br>- Adobe Photoshop
Tile Pyramid creation	- Openzoom / Deepzoom<br>- **Zoomify**<br>- **Image Composite Editor**<br>- scikit-image: pyramid.gaussian<br>- OpenCV: pyrDown()
View	- **OpenSeadragon**<br>- **Zoomify**<br>- OpenLayers<br>- Panellum (Matthew Petroff)
Annotations / Overlays / Extensions	- *OpenLayers*<br>- ***OpenSeadragon Plugins***<br>- *Zoomify*

Journal of Cell Science • Supplementary information

Table S2. html examples for websites using different visualisation approaches, https://github.com/wohlmann/2024_EMMA_BOXES

Description	html template
html example for a website with a **dzi and OpenSeadragon-based** container as well as a **floating scalebar**, https://github.com/wohlmann/2024_EMMA_BOXES	<!doctype html><br><html lang="en"><br><head><br><meta charset="UTF-8"><br><style type="text/css"> #*YOURID* { width: 90%; height: 90%; } </style><br></head><br><body><br><div id="*YOURID*"></div><br><script src="*openseadragon/*openseadragon.min.js"></script><br><script src="*openseadragon/*openseadragon-scalebar.js"></script><br><script><br>var viewer = OpenSeadragon({<br>id: '*YOURID*',<br>prefixUrl: 'openseadragon/images/',<br>tileSources: '*dzi/YOURID.dzi*'<br>});<br>viewer.scalebar({<br>pixelsPerMeter: *YOURVALUE*,<br>xOffset: 20,<br>yOffset: 10,<br>});<br></script><br></body><br></html>
html example for a website with a **single Zoomify-based container**, https://github.com/wohlmann/2024_EMMA_BOXES	<!doctype html><br><html lang="en"><br><head><br><meta http-equiv="Content-Type" content="text/html; charset=utf-8" /><br><script type="text/javascript" src="Zoomify.js"></script><br><style type="text/css"> #Container { width:95%; height:95%; margin:auto; } </style><br><script type="text/javascript"> Z.showImage("myContainer", "*FOLDER*"); </script><br></head><br><body bgcolor=#000000><br><br><br><div id="Container"></div><br><br><br></body><br></html>

html example for a website with **two Zoomify-based containers**, https://github.com/wohlmann/2024_EMMA_BOXES

```
<!doctype html>
<html lang="en">
        <head>
                <meta http-equiv="Content-Type" content="text/html; charset=utf-8" />
                <script type="text/javascript" src="Zoomify.js"></script>
                <style> html, body { justify-content:center; height: 95%; width: 95%;
                margin: 5px; </style>
                <style type="text/css"> #myContainer1 { float:left; width:49%; height:90%;
                margin:auto; } </style>
                <style type="text/css"> #myContainer2 { float:left; width:49%; height:90%;
                margin:auto; } </style>
                <script type="text/javascript">
                        Z.showImage("myContainer1", "FOLDER1");
                        Z.showImage("myContainer2", "FOLDER2");
                </script>
        </head>
        <body bgcolor="#000000">
                <br>
                <div id="myContainer1"></div>
                <div id="myContainer2"></div>
                <br>
        </body>
</html>
```

Journal of Cell Science • Supplementary information

html example for a website with an **OpenSeadragon container with floating scalebar and 11 switchable overlays,** https://github.com/wohlmann/2024_EMMA_BOXES

```
<!DOCTYPE html>
<html lang="en">
<head>
        <meta charset="UTF-8">
        <style>html, body {height: 98%; width: 100%; margin: 2px; background: #000000;} #openseadragon-viewer {width: 95%;
        height: 90%; margin: auto; border: 1px solid #696969; margin-bottom: 5px;} #buttons-container {text-align: center;
        display: flex; justify-content: center; flex-wrap: wrap;} #overlay-toggle {margin-top: 5px;padding: 3px 5px;display:
        block;box-sizing: border-box;} .overlay-toggle.active {box-shadow: inset 0 0 0 2px #dc0404;border: none;}
        </style>
</head>
<body>
  </div>
  <div id="openseadragon-viewer"></div>
  <div id="buttons-container"></div>
  <script src="openseadragon/openseadragon.js"></script>
  <script src="openseadragon/openseadragon-scalebar.js"></script>
  <script>   var viewer = OpenSeadragon({
                id: "openseadragon-viewer", prefixUrl: "openseadragon/images/",
                tileSources: [{
                type: "zoomifytileservice",width: 32343,height: 14888,tilesUrl:
                2018008_029_2018008B_H4_ser1_col_2/",tileSize: 256, fileFormat: "jpg" }],showNavigator: true,
                navigatorPosition: "ABSOLUTE", navigatorTop: "10px", navigatorLeft: "20px", navigatorHeight:
                "45%",navigatorWidth: "45%", navigatorAutoFade: true, wrapHorizontal: false, wrapVertical: false,
                zoomPerScroll: 1.2, minZoomImageRatio: 0.5, showNavigationControl: false
        });
        var overlaysConfig = [{
                name: "Basal lamina, Neural Tube", path: "OVERLAYS/001/", color: "#b58fee", fontColor: "#000000",
                fontWeight: "bold", opa: "0.4"},{name: "Lumen Vasculature", path: "OVERLAYS/002 /", color: "#ffc2c2",
                fontColor: "#000000", fontWeight: "bold", opa: "0.2"},{name: "Endothelium", path: "OVERLAYS/003 /",
                color: "#fc8e8e", fontColor: "#000000", fontWeight: "bold", opa: "0.2"},{name: "Marginal Zone", path:
                "OVERLAYS/004 /", color: "#88c6a5", fontColor: "#000000", fontWeight: "bold", opa: "0.2"},{name:
                "Ventricular Zone, Nuclei", path: "OVERLAYS/005 /", color: "#dfa9d6", fontColor: "#000000", fontWeight:
                "bold", opa: "0.2"},{name: "Ventricular Zone, Cytoplasm", path: "OVERLAYS/006/", color: "#9391fa",
                fontColor: "#000000", fontWeight: "bold", opa: "0.2"},{name: "B16-Tumor", path: "OVERLAYS/007 /", color:
                "#f1f69f", fontColor: "#000000", fontWeight: "bold", opa: "0.2"},{name: "Neural Tube", path:
                "OVERLAYS/008 /", color: "#c6d6ee", fontColor: "#000000", fontWeight: "bold", opa: "0.2"},        {name:
                "Muscle", path: "OVERLAYS/009 /", color: "#f3cfcf", fontColor: "#000000", fontWeight: "bold", opa:
                "0.2"},{name: "unidentified", path: "OVERLAYS/010 /", color: "#9ffc6c", fontColor: "#000000", fontWeight:
                "bold", opa: "0.15"}, {name: "Orientation LM", path: "OVERLAYS/011 /", color: "#cfcfcf", fontColor:
                "#000000", fontWeight: "bold", opa: "1"
        }];
        var tiledImages = [];
        viewer.addHandler("open", function()
                {overlaysConfig.forEach(function(overlayConfig, index) {
                        var button = document.createElement("button"); button.innerHTML = overlayConfig.name;
                        button.style.border = "none"; button.style.marginRight = "5px";button.style.backgroundColor =
                        overlayConfig.color; button.style.fontWeight = overlayConfig.fontWeight; button.style.color =
                        overlayConfig.fontColor; button.classList.add("overlay-toggle");
                        button.onclick = function() {
                                var tile = tiledImages[index];
                                var isActive = tile.getOpacity() > 0;
                                if (isActive) {tile.setOpacity(0);
                                        button.classList.remove('active'); button.style.border = "none";
                                }else {
                                tile.setOpacity(parseFloat(overlayConfig.opa));button.classList.add('active');
                                                button.style.border = "";
                                }
                         };
                        document.getElementById("buttons-container").appendChild(button);
                        viewer.addTiledImage({
                                tileSource: { type: "zoomifytileservice", width: 32343, height: 14888, tileSize: 256,
                                fileFormat: "png", tilesUrl: overlayConfig.path}, opacity: 0, success: function(event)
                                {tiledImages[index] = event.item; }
                        });
                });
                viewer.scalebar({ type: "MICROSCOPY", pixelsPerMeter: 223495200, xOffset: 20, yOffset: 25,
                stayInsideImage: true,          location: OpenSeadragon.ScalebarLocation.BOTTOM_RIGHT, color:
                "rgba(0,0,0,255)", fontColor: "rgba(0,0,0,255)",
                backgroundColor: 'rgba(255,255,255,0)', fontSize: 'large', barThickness: 4, minWidth: "200px",
                });
        });
  </script>
</body>
</html>
```

Journal of Cell Science • Supplementary information

Table S3. List of Links for the mentioned software, names in bolt.

List of Links for the mentioned software, names in bolt.	https://bio3d.colorado.edu/**SerialEM**/<br>https://imagej.net/plugins/**bigstitcher**/<br>https://**openseadragon**.github.io/<br>https://**openseadragon**.github.io/#**plugins**)<br>https://github.com/usnistgov/**OpenSeadragonScalebar**<br>https://github.com/**openseadragon**/svg-**overlay**<br>https://github.com/Emigre/**openseadragon-annotations**<br>https://github.com/usnistgov/**OpenSeadragonFiltering**<br>https://**openlayers**.org/<br>https://**openlayers**.org/**3rd-party**/<br>https://**openlayers**.org/en/latest/examples/**measure**.html<br>https://www.**openzoom**.org/<br>https://**annotorious**.github.io/<br>http://**paperjs**.org/<br>http://**fabricjs**.com/<br>https://github.com/openzoom/**deepzoom**.py<br>https://**opencv**.org/<br>https://**scikit**-image.org/<br>https://github.com/mpetroff/**pannellum**<br>https://**hugin**.sourceforge.io/<br>https://www.**panotools**.org/<br>https://www.microsoft.com/en-us/research/project/**image-composite-editor**/<br>https://www.**gimp**.org<br>https://www.**zoomify**.com<br>https://www.adobe.com/products/**photoshop**.html

Journal of Cell Science • Supplementary information
