# Supplementary material for: Expanding the field of view – a simple approach for interactive visualisation of electron microscopy data
Source: J Cell Sci. 2024 Oct 23;137(20):jcs262198. doi: 10.1242/jcs.262198 (PMC11529876; doi:10.1242/jcs.262198)
Supplement: Supplementary file 1 [file joces-137-262198_review_history.pdf]

Expanding the field of view - a simple approach for interactive visualisation of electron microscopy data.

Jens Wohlmann
DOI: 10.1242/jcs.262198

Editor: Lucy Collinson

Review timeline

Original submission:	11 April 2024
Editorial decision:	22 July 2024
First revision received:	1 August 2024
Editorial decision:	9 September 2024
Second revision received:	11 September 2024
Accepted:	12 September 2024

Original submission

First decision letter

MS ID#: JOCES/2024/262198

MS TITLE: Expanding the field of view: A simple approach for interactive visualisation of electron microscopy data at continuous magnification.

AUTHORS: Jens Wohlmann
ARTICLE TYPE: Tools and Resources

We have now reached a decision on the above manuscript.

To see the reviewers' reports and a copy of this decision letter, please go to: https://submit-jcs.biologists.org and click on the 'Manuscripts with Decisions' queue in the Author Area. (Corresponding author only has access to reviews.)

As you will see, the reviewers raise a number of substantial criticisms that prevent me from accepting the paper at this stage. They suggest, however, that a revised version might prove acceptable, if you can address their concerns. If you think that you can deal satisfactorily with the criticisms on revision, I would be pleased to see a revised manuscript. We would then return it to the reviewers.

Please ensure that you clearly highlight all changes made in the revised manuscript. Please avoid using 'Tracked changes' in Word files as these are lost in PDF conversion.

I should be grateful if you would also provide a point-by-point response detailing how you have dealt with the points raised by the reviewers in the 'Response to Reviewers' box. Please attend to all of the reviewers' comments. If you do not agree with any of their criticisms or suggestions please explain clearly why this is so.

Reviewer 1

Advance summary and potential significance to field

© 2024. Published by The Company of Biologists under the terms of the Creative Commons Attribution License (https://creativecommons.org/licenses/by/4.0/).

The article makes a good case for the importance of visualisation across magnification scales, specifically in the case of 2D EM data. It describes methodology to implement such visualisations, and provides a suggested pipeline to preprocess data in order for end users to host these visualisations on their own web infrastructure.

Comments for the author

While clear in its problem description and possible specific solution, the manuscript misses considerable “prior art” in most aspects of the pipeline. Although based on closed-source software, Nanotomy (http://www.nanotomy.org/) has provided scalable 2D EM visualisation for a long time. More recently initiatives such as OpenOrganelle (https://openorganelle.janelia.org/) provide such functionality for 3D data (using an open source approach which would also work in 2D). Given these exist, it is less clear what novelty is provided by the approach suggested. Use of OpenSeaDragon for large histology images is common in the light microscopy world, and although there are, as the author indicates, some particular requirements around stitching 2D EM images, that is a more specific problem

While the author suggests that enabling sharing of data alongside manuscript figures is an aim, there are considerable challenges involved in this process that are left unresolved. How does this operate at scale?
How should the manuscript be linked to the interactive figure? Will the hosting institution commit to long term data preservation? The long term history of the JCB dataviewer suggests that these are not trivial challenges (https://rupress.org/jcb/pages/jcb-dataviewer)!

The manuscript also seems to suggest that the primary purpose of image segmentation (including via deep learning) is to isolate and analyse single isolated events, or produce visualisations. Frequently the purpose of semantic segmentation is to allow large scale quantisation of 2D or 3D EM data to enable population level measurements of numerical descriptors that go considerably beyond what is possible with stereology, which is why, for example, large scale modern connectomics is extremely reliant on segmentation.

Finally, although the proposed pipeline makes sense at a high level, it is not clear what its target audience is.
If it is intended to be used by research biologists / imaging scientists in general, some steps (e.g. hand-editing HTML) are non-trivial. If intended for expert users, or for use on large scale compute systems reliance on Windows-only interactive editing tools will cause problems.

Reviewer 2

Advance summary and potential significance to field

The author presents an interactive web-compatible visualisation strategy for multi-resolution rendering of electron microscopy data.

The topic of this article is very timely, and I think it is a great idea to place QR codes into the published figures that lead to an interactive multi-resolution view of the data. I greatly enjoyed opening those URLs and smoothly browsing the data both on my computer and smart phone.

I think having a solution like this becoming more mainstream would greatly improve the way (EM) image data is published!

Comments for the author

My main concern is that, in my opinion, this article should discuss in more detail alternative efforts towards the same aim, i.e. creating URLs for interactive browsing of large image data. Specially, the community has been working hard to implement the following OME-Zarr based workflow for web-compatible multi-scale image data sharing and viewing; see this publication for an overview: https://link.springer.com/article/10.1007/s00418-023-02209-1

© 2024. Published by The Company of Biologists under the terms of the Creative Commons Attribution License (https://creativecommons.org/licenses/by/4.0/).

1. Publish the data on the BioImage Archive (https://www.ebi.ac.uk/bioimage-archive/)

2. The BioImage Archive will automatically convert the data to the 3-D chunked multi-resolution OME-Zarr file format, host this on S3 object stores and provide the corresponding URL. For example see: https://www.ebi.ac.uk/bioimage-archive/galleries/vem.html; click on one of the data sets and then click the "view" button, this yields interactive browsing URLs such as: https://www.ebi.ac.uk/bioimage-archive/galleries/EMPIAR-10442/IM1.html

3. As detailed in https://link.springer.com/article/10.1007/s00418-023-02209-1 the provided OME-Zarr URLs can be rendered in many viewers, including web viewers but also common desktop platforms such as Fiji and Napari.

Another example URL for interactive browsing of 8 TB 3D EM volume raw data in neuroglancer:
https://neuroglancer-demo.appspot.com/#!%7B%22dimensions%22:%7B%22z%22:%5B2.5e-8%2C%22m%22%5D%2C%22y%22:%5B1e-8%2C%22m%22%5D%2C%22x%22:%5B1e-8%2C%22m%22%5D%7D%2C%22position%22:%5B5976.87353515625%2C14190.419921875%2C17355.09765625%5D%2C%22crossSectionScale%22:31.543553816683286%2C%22projectionOrientation%22:%5B-0.33406034111976624%2C0.1957729160785675%2C-0.0033079788554459 81%2C0.921990096569061 3%5D%2C%22projectionScale%22:32768%2C%22layers%22:%5B%7B%22type%22:%22image%22%2C%22source%22:%22zarr://https://s3.embl.de/i 2k-2020/platy-raw.ome.zarr%22%2C%22tab%22:%22source%22%2C%22name%22:%22platy-raw.ome.zarr%22%7D%5D%2C%22selectedLayer%22:%7B%22visible%22:true%2C%22layer%22:%22platy-raw.ome.zarr%22%7D%2C%22layout%22:%224panel%22%2C%22helpPanel%22:%7B%22visible%22:true%7D%7D

In my opinion this workflow has several good features:

1. The user does not need to install any software to make the data publicly available, all is done at the BioImage Archive.
2. The user does not need to self host any data.
3. It anyway is good practice to publish data in an archive, thus publishing data at the BioImage Archive is useful, not only for web rendering.
4. The OME-Zar represents a lossless compressed version of the raw data that is fully suitable for analysis.
5. This approach also supports 3-D, time-lapse and multi-channel data.
6. Some of viewers, such as the above neuroglancer URL, capture the complete viewer state including rotations and zoom level; this is great for sharing specific views of the same data with collaborators

In addition to the BioImage Archive also institutions such as Janelia Research Campus have adopted similar strategies. See https://openorganelle.janelia.org/ where URLs like the below enable interactive browsing:

https://neuroglancer-demo.appspot.com/#!%7B%22dimensions%22:%7B%22x%22:%5B1e-9%2C%22m%22%5D%2C%22y%22:%5B1e-9%2C%22m%22%5D%2C%22z%22:%5B1e-9%2C%22m%22%5D%7D%2C%22position%22:%5B15598.5%2C5998.5%2C10090.5%5D%2C%22crossSectionScale%22:50%2C%22projectionScale%22:65536%2C%22layers%22:%5B%7B%22type%22:%22image%22%2C%22source%22:%22zarr://s3://janelia-cosem-datasets/jrc_mus-granule-neurons-3/jrc_mus-granule-neurons-3.zarr/recon-2/em/fibsem-int16%22%2C%22tab%22:%22rendering%22%2C%22opacity%22:1%2C%22blend%22:%22additive%22%2C%22shader%22:%22#uicontrol%20invlerp%20normalized%28range=%5B9744%2C%206364%5D%2C%20window=%5B4674%2C%2011434%5D%29%5Cn#uicontrol%20vec3%20color%20color%28default=%5C%22white%5C%22%29%5Cnvoid%20main%28%29%7BemitRGB%28color%20%2A%20normalized%28%29%29%3B%7D%22%2C%22name%22:%22fibsem-int16%22%7D%5D%2C%22selectedLayer%22:%7B%22visible%22:true%2C%22layer%22:%22fibsem-int16%22%7D%2C%22crossSectionBackgroundColor%22:%22#000000%22%2C%22layout%22:%224panel%22%7D

© 2024. Published by The Company of Biologists under the terms of the Creative Commons Attribution License (https://creativecommons.org/licenses/by/4.0/).

Having said this, I could also see that the approach that is presented by the author might have some advantages over the OME-Zarr strategy. I am very much looking forward to read the author's opinions and comparisons!

First revision

Author response to reviewers' comments

I would like to thank the reviewers for their valuable criticism of the submitted manuscript. Below, I am addressing the criticism and the points raised individually.

Reviewer 1 (red corrections in the main document):

1. *While clear in its problem description and possible specific solution, the manuscript misses considerable "prior art" in most aspects of the pipeline. Although based on closed-source software, Nanotomy (http://www.nanotomy.org/) has provided scalable 2D EM visualisation for a long time. More recently, initiatives such as OpenOrganelle (https://openorganelle.janelia.org/) provide such functionality for 3D data (using an open source approach which would also work in 2D). Given these exist, it is less clear what novelty is provided by the approach suggested.*

In our perspective, the novelty of the proposed approach lies in its simplicity, the open-source and low-tech solution and the straightforward implementation together with the flexible adaptability from a small local hosting to a large-scale implementation in e.g. online publications without the need for major modifications. These factors are addressed in e.g. **line 382-384, 388-390** and **426-428**. In addition, the approach's focus on 2D EM data and the optional workflow specifically for such data is, to our knowledge, not found in the literature.

Adapted changes in the manuscript:

- A sentence addressing prior solutions outside of the 3D realm, mentioning "nanotomy" was added. (**line 102-104**)
- The example "OpenOrganelle" was added to the pre-existing paragraph on prior solutions visualising 3D data. (**line 106**) and in a paragraph on alternative solutions (line 85)
- In the discussion (**line 377**) a qualifying statement ("open source") was added to make the sentence more clear.
- Two paragraphs to alternative approaches are added (**line 109-118, line 437-465**),

2. Use of Openseadragon for large histology images is common in the light microscopy world, and although there are, as the author indicates, some particular requirements around stitching 2D EM images, that is a more specific problem

In general, a prerequisite for applying such a visualisation strategy is a useful base dataset, and given the under-representation of methods suitable for 2D EM data, it seemed important to provide a workflow for generating such data, hence the chapter on processing and stitching EM data. This aspect is related to the target audience of the publication as discussed in **point 5**.

Adapted changes in the manuscript:

- The information about the common use of Openseadragon for histology is added to the introducing sentence. (**line 233-234**)

3. *While the author suggests that enabling sharing of data alongside manuscript figures is an aim, there are considerable challenges involved in this process that are left unresolved. How does this operate at scale? How should the manuscript be linked to the interactive figure? Will the hosting institution commit to long term data preservation? The long term history of the JCB dataviewer suggests that these are not trivial challenges (https://rupress.org/jcb/pages/jcb-dataviewer)!*

© 2024. Published by The Company of Biologists under the terms of the Creative Commons Attribution License (https://creativecommons.org/licenses/by/4.0/).

Undoubtedly, long-term storage of (primary) data such as the solution we propose is a difficult issue. However, it is obviously of utmost value to do so and fortunately improvements in IT infrastructure allow for more and more accessible solutions from a hardware point of view. Relevant examples are the massive increase in storage capacity and faster Ethernet connections. Projects such as OpenOrganelle or the rebirth of the JCB Dataviewer as 'BioStudies' in 2018 illustrate this well. The solution we propose has the advantage that it only requires relatively small datasets and low technical requirements compared to multi-omics or volume EM data. This enables local hosting at the institution or on any web space. For publications, the publisher should ideally host the data, as there will be a repository for the publication already, often containing video files larger than an EM data set for a whole cell, for example.

Adapted changes in the manuscript:

- Cloud services and publisher hosting are added as options in **line 130**.

4. *The manuscript also seems to suggest that the primary purpose of image segmentation (including via deep learning) is to isolate and analyse single isolated events, or produce visualisations. Frequently the purpose of semantic segmentation is to allow large scale quantisation of 2D or 3D EM data to enable population level measurements of numerical descriptors that go considerably beyond what is possible with stereology, which is why, for example, large scale modern connectomics is extremely reliant on segmentation.*

We can understand the scepticism towards stereology compared to the intuitive approach of volume quantification based on segmented 3D-EM data. It is admittedly counterintuitive to imagine that a few thin slices of a tissue or organ could provide an accurate image or even precise quantification of its structures in 3D - after all, the estimations are based on an incredibly small fraction. However, this is indeed the case and it requires just a minute fraction of the effort and computation power required for segmentation based quantification. It is really surprising how underused stereological methods are, but there is currently a trend towards their revival in the field of image analysis. Two publications cited in the manuscript address this particular issue of 3D quantification by stereology versus segmentation (Lucocq et al., 2015, Mayhew et al., 2015). A remarkable demonstration of the power of stereology for such questions is the analysis of the cellular composition of the human brain (von Bartheld et al., 2018) based on design-based stereology using the isotropic fractionator technique (Gundersen et al., 1987). Hence, the measurement of numerical population descriptors is indeed simpler and faster with stereological methods, which can also be applied to subsamples of volume datasets, allowing a comparison of results. In contrast, connectomics deals with (often large numbers of) individual structures (e.g. neurons) and their individual descriptors - if these identities are to be preserved in the presence of multiple entities, a segmentation based approach would be the preferred method.

Lucocq, J. M.; Mayhew, T. M.; Schwab, Y.; Steyer, A. M.; Hacker, C., Systems biology in 3D space--enter the morphome. *Trends Cell Biol* **2015,** *25* (2), 59-64.
Mayhew, T. M.; Lucocq, J. M., From gross anatomy to the nanomorphome: stereological tools provide a paradigm for advancing research in quantitative morphomics. *J Anat* **2015,** *226* (4), 309-21.
von Bartheld, C. S., Myths and truths about the cellular composition of the human brain: A review of influential concepts. *J Chem Neuroanat* **2018,** *93*, 2-15.
Gundersen, H. J.; Jensen, E. B., The efficiency of systematic sampling in stereology and its prediction. *J Microsc* **1987,** *147* (Pt 3), 229-63.

Adapted changes in the manuscript:

- Added the exception of connectomics to the stereology paragraph (**line 358**)

5. *Finally, although the proposed pipeline makes sense at a high level, it is not clear what its target audience is. If it is intended to be used by research biologists / imaging scientists in general, some steps (e.g. hand- editing HTML) are non-trivial. If intended for expert users, or for use on large scale compute systems, reliance on Windows-only interactive editing tools will cause problems.*

Our primary target audience is research biologists who use electron microscopy to understand their particular questions or who teach electron microscopy. The primary aim and the current application of

© 2024. Published by The Company of Biologists under the terms of the Creative Commons Attribution License (https://creativecommons.org/licenses/by/4.0/).

the methodology is within online or offline discussions, in teaching or in publications. While manual editing of HTML might be a new aspect for research biologists, it is common for most of them to use some level of coding in the respective research projects - be it R, ImageJ macro, Python or similar. The html requirements for using our proposed method are really minimal and arguably easier to understand than most image analysis pipelines. In addition, the manuscript is accompanied by ready-to-use examples with instructions on how to customise them and versions for different scenarios. Focusing on a possible easy implementation and minimal technological effort, our "simplest" approach uses graphic user interface (GUI)-based and windows software – however, for expert users the same methodology can be applied independent of the operation system through python pipelines e.g. based on the scikit-image packages. Useful tools in general are summarised in a supplemental table (attached below) with the respective text passage being in **line 509 - 513**.

Step	Software
Record	- Microscope manufacturer<br>- SerialEM
Align	- Microscope manufacturer<br>- Big Stitcher<br>- Hugin<br>- **Image Composite Editor**<br>- SerialEM
Correct if necessary (e.g. in ImageJ)	- polynomial shading correction<br>- *Rolling ball background subtraction*<br>- Fourier filtering
Stitch / Project	- Microscope manufacturer<br>- Big Stitcher<br>- Hugin<br>- Panotools<br>- GIMP<br>- **Image Composite Editor**<br>- Adobe Photoshop
Tile Pyramid creation	- Openzoom / Deepzoom<br>- **Zoomify**<br>- **Image Composite Editor**<br>- scikit-image: pyramid.gaussian<br>- OpenCV: pyrDown()
View	- OpenSeadragon<br>- **Zoomify**<br>- OpenLayers<br>- Panellum (Matthew Petroff)
Annotations / Overlays / Extensions	- OpenLayers<br>- ***OpenSeadragon Plugins***<br>- Zoomify

Overview of a selection of useable software tools, the simplest solution is printed in bold and optional steps in italics

Reviewer 2 (green corrections in the main document):

For better overview the URLs are replaced with "**URL**".

My main concern is that, in my opinion, this article should discuss in more detail alternative efforts towards the same aim, i.e. creating URLs for interactive browsing of large image data. Specially, the community has been working hard to implement the following OME-Zarr based workflow for web-compatible multi-scale image data sharing and viewing; see this publication for an overview: ***URL***

1. *Publish the data on the BioImage Archive (**URL**)*
2. *The BioImage Archive will automatically convert the data to the 3-D chunked multi-resolution OME-Zarr file format, host this on S3 object stores and provide*

© 2024. Published by The Company of Biologists under the terms of the Creative Commons Attribution License (https://creativecommons.org/licenses/by/4.0/).

*the corresponding URL. For example see: **URL**; click on one of the data sets and then click the "view" button, this yields interactive browsing URLs such as: **URL***

*3. As detailed in **URL** the provided OME-Zarr URLs can be rendered in many viewers, including web viewers but also common desktop platforms such as Fiji and Napari.*

*Another example URL for interactive browsing of 8 TB 3D EM volume raw data in neuroglancer: **URL***

In my opinion this workflow has several good features:

1. The user does not need to install any software to make the data publicly available, all is done at the BioImage Archive.

2. The user does not need to self host any data.

3. It anyway is good practice to publish data in an archive, thus publishing data at the BioImage Archive is useful, not only for web rendering.

4. The OME-Zar represents a lossless compressed version of the raw data that is fully suitable for analysis.

5. This approach also supports 3-D, time-lapse and multi-channel data.

6. Some of viewers, such as the above neuroglancer URL, capture the complete viewer state including rotations and zoom level; this is great for sharing specific views of the same data with collaborators

*In addition to the BioImage Archive also institutions such as Janelia Research Campus have adopted similar strategies. See **URL** where URLs like the below enable interactive browsing: **URL***

Having said this, I could also see that the approach that is presented by the author might have some advantages over the OME-Zarr strategy. I am very much looking forward to read the author's opinions and comparisons!

While the goal of creating a common file format and centralised data stores that integrate this data to put it into context is a great and noble idea, the scope of our present attempt is a much smaller one. Especially in the early stages of projects or when adapting methodologies, browsing multiscale data can have immense value to the discussion. Information from different scales or areas of the sample can be of great value when consulting experts on the topic at hand or in discussions with co- operation partners, which often take place online. At this stage of the project, however, the data may not yet fulfil the standards of a repository or the sample may not be suitable for publication due to its nature, as it could be patient material or the commercial interests of cooperation partners must be taken into account. This is where self-hosting can be advantageous; in more delicate cases, it can be done internally in an institution or even just on a local computer such as a laptop. Whilst self- hosting can generally be seen as a disadvantage, it also has some upsides. First and foremost, no third party service is required and changes to terms and conditions are unlikely - however this is only possible on a smaller scale or perhaps at institute level. At this level, dedicated datasets for teaching and specific tasks can be realised that would not be of interest on a larger scale. Our goal is a simple solution that can be flexibly adapted to individual needs - especially in the field of classical electron microscopy (outside the VolumeEM and Cryo-EM community) the development towards digital solutions seems to be delayed. One reason for this could be the difficulty in finding capable open source tools for data processing and image stitching - our goal is to provide a simple but compatible workflow that is easy to use and understand in order to lower the barrier for such developments.
While the OME-Zarr format is certainly a great idea, we think that data conversion can be seen as another hurdle. In addition, the large number of features and possibilities in most image repositories can be overwhelming when only a simple task is required, and this is often the case with 2D EM data. The field of 3D EM seems to be more open and interested in digital solutions - probably because this field is often technology-driven and image analysis and processing are essential parts of the workflows - the same is true for cryo-EM. However, we believe that there is immense potential in using classical EM approaches - especially in combination with stereology.
In terms of advanced features such as saving the region and zoom level of a shared dataset and incorporating them into a URL, this is also possible in Openseadragon. The use of Openseadragon generally holds huge potential, as a wealth of additional features are already implemented and further options are available through the open source community. One of the hopes we have is that the application of this visualisation strategy in publications will become a reality.

The comparison with alternative and already existing solutions as well as with the current and promising attempts of the bio imaging community to find a common file format (OME-Zarr) are important aspects

© 2024. Published by The Company of Biologists under the terms of the Creative Commons Attribution License (https://creativecommons.org/licenses/by/4.0/).

that we were unfortunately not aware of. The criticism is greatly appreciated and several adjustments have been incorporated into the manuscript. (**line 109-118, line 121-122 and line 437-465**)

Second decision letter

MS ID#: JOCES/2024/262198

MS TITLE: Expanding the field of view: A simple approach for interactive visualisation of electron microscopy data.

AUTHORS: Jens Wohlmann
ARTICLE TYPE: Tools and Resources

We have now reached a decision on the above manuscript.

To see the reviewers' reports and a copy of this decision letter, please go to: https://submit-jcs.biologists.org and click on the 'Manuscripts with Decisions' queue in the Author Area. (Corresponding author only has access to reviews.)

As you will see, the reviewers gave favourable reports but raised some minor additional critical points that will require amendments to your manuscript. I hope that you will be able to carry these out as soon as possible because I would like to be able to accept your paper.

Please ensure that you clearly highlight all changes made in the revised manuscript. Please avoid using 'Tracked changes' in Word files as these are lost in PDF conversion.

I should be grateful if you would also provide a brief response detailing how you have dealt with the points raised by the reviewers in the 'Response to Reviewers' box. Please attend to all of the reviewers' comments. If you do not agree with any of their criticisms or suggestions please explain clearly why this is so.

Reviewer 1

Advance summary and potential significance to field

The updates to the manuscript address my concerns about the work. The described approach is clearly likely to be of benefit to both individual researchers and groups that wish to share their data using a straightforward, self-hosted approach. Adding the consideration of existing alternatives and the unique features of the proposed system strengthen the manuscript and, in my opinion, render is suitable for publication in JCS.

Comments for the author

As described above, the changes and updates to the manuscript address my concerns, and the author is to be congratulated on a useful approach and valuable work.

Reviewer 2

Advance summary and potential significance to field

See first review.

Comments for the author

© 2024. Published by The Company of Biologists under the terms of the Creative Commons Attribution License (https://creativecommons.org/licenses/by/4.0/).

I would like to thank the author for adding a discussion of OME-Zarr based solutions to the article. In fact, I got now a bit confused as to how much of an OME-Zarr based discussion should actually go into this article. Given that the author is not an OME-Zarr expert I do not think it is reasonable to require too many details. In addition, as I understand it and as the article title suggests the main point of the article is to propose an OpenSeaDragon based workflow, as detailed in Table 1.

I have reread the article with this in mind and I found that a general issue of the article is that it appears as a mix of a review of existing methods and concrete recommendations what to do. For example, the section “Image stitching of EM-data” (line 141-177) is a subsection of the “Results” section of the article. However I could not find any concrete “result” in a sense of a concrete workflow that could be implemented; rather this section reviews some existing tools that are related to the stitching of image data. I think the article could strongly benefit from making a clearer distinction from what is a review of the current field and where the actual novel recommended workflow is explained. From an editorial point of view I don’t know whether the article could contain both a “Review” section and a “Results” section? If so, I would recommend putting into the results section only text that directly relates to Table 1, such that readers that are not interested in a review of existing methods could directly jump to the part where they can learn how to implement the novel OpenSeaDragon based method. I would then also recommend adding more details on how to implement the novel workflow. For instance adding details for how to use the Deepzoom python based approach would be very valuable to make the new workflow accessible to users that do not have a windows computer. In addition, more hands-on information on how to practically share the data with remote collaborators would be very useful (e.g. how to set up a github repository to host the data).

I feel that if the article would be restructured/rewritten along those lines, it could be easier to decide how much and where details about other methods, such as OME-Zarr based workflows, would need to be mentioned.

Detailed comments:

Line 99ff:
Thank you for adding the discussion of the OME-Zarr based solution, starting at line 437. I however think that already here in the introduction part it should at least be mentioned. Maybe you could add a sentence like: “Another relevant interactive solution is based on the new OME-Zarr image file format (Moore et al.); this will be discussed in more detail further below.”

Line 122: “without the need for a dedicated software or format.”
I don’t think that this is a fair assessment, because the current instructions need the (dedicated) Microsoft ICE msi software and the raw data is converted to the (dedicated) DZI format. This is, in my view, as complex and arguable even less straightforward than, e.g., using the Fiji/ImageJ software (File › Save As › HDF5/N5/Zarr/OME-NGFF …) to convert the data to OME-Zarr format. In addition maybe even easier than Fiji, one can create OME-Zarr using this tool: https://forum.image.sc/t/ngff-converter-2-0-released-convert-bioformats-to-ome-ngff-ome-tiff/96102.

Line 378: As a citation for the Fiji plugin MoBIE, this dedicated publication seeme more suitable than Vergara et al.: https://www.nature.com/articles/s41592-023-01776-4

Line 456: “format itself could be implemented but the visualisation it is based on repositories”
I don’t think this statement is correct like this. There are no repositories needed for the visualisation. As mentioned in my first review, on can construct an URL (see below) uses the open-access neuroglancer (https://github.com/google/neuroglancer) visualisation tool to open a data set (https://s3.embl.de/i2k-2020/platy-raw.ome.zarr) that is hosted on a publicly accessible S3 server at EMBL. Someone just happened to put the data there and there are no curated repositories involved at all. Anyone with access to an S3 object store at their institution or, e.g., at Amazon can put any data there and can use any of the various open-access visualisation tools mentioned in https://link.springer.com/article/10.1007/s00418-023-02209-1 to view the data without the need for any curation or review. For sensitive data some S3 object stores also offer secret/public key protected access

© 2024. Published by The Company of Biologists under the terms of the Creative Commons Attribution License (https://creativecommons.org/licenses/by/4.0/).

(https://min.io/docs/minio/linux/administration/identity-access-management/minio-user-management.html).

Neuroglancer URL to visualise a S3 hosted OME-Zarr image:
https://neuroglancer-demo.appspot.com/#!%7B%22dimensions%22:%7B%22z%22:%5B2.5e-8%2C%22m%22%5D%2C%22y%22:%5B1e-8%2C%22m%22%5D%2C%22x%22:%5B1e-8%2C%22m%22%5D%7D%2C%22position%22:%5B5976.87353515625%2C14190.419921875%2C17355.09765625%5D%2C%22crossSectionScale%22:31.543553816683286%2C%22projectionOrienta tion%22:%5B-0.3340603411976624%2C0.1957729160785675%2C-0.0033079788554459812C0.921990096569061 3%5D%2C%22projectionScale%22:32768%2C%22layers%22:%5B%7B%22type%22:%22image%22%2C%22source%22:%22zarr://https://s3.embl.de/i2k-2020/platy-raw.ome.zarr%22%2C%22tab%22:%22source%22%2C%22name%22:%22platy-raw.ome.zarr%22%7D%5D%2C%22selectedLayer%22:%7B%22visible%22:true%2C%22layer%22:%22platy-raw.ome.zarr%22%7D%2C%22layout%22:%224panel%22%2C%22helpPanel%22:%7B%22visible%22:true%7D%7D

Second revision

Author response to reviewers' comments

I would like to thank the reviewers for their valuable criticism of the submitted manuscript. Below, I am addressing the criticism and the points raised individually.

Reviewer 2 (green corrections in the main document):

For better overview the URLs are replaced with "**URL**".

I would like to thank the author for adding a discussion of OME-Zarr based solutions to the article. In fact, I got now a bit confused as to how much of an OME-Zarr based discussion should actually go into this article. Given that the author is not an OME-Zarr expert I do not think it is reasonable to require too many details. In addition, as I understand it and as the article title suggests, the main point of the article is to propose an OpenSeaDragon based workflow, as detailed in Table 1.

I have reread the article with this in mind and I found that a general issue of the article is that it appears as a mix of a review of existing methods and concrete recommendations what to do. For example, the section "Image stitching of EM-data" (line 141-177) is a subsection of the "Results" section of the article. However I could not find any concrete "result" in a sense of a concrete workflow that could be implemented; rather this section reviews some existing tools that are related to the stitching of image data. I think the article could strongly benefit from making a clearer distinction from what is a review of the current field and where the actual novel recommended workflow is explained. From an editorial point of view I don't know whether the article could contain both, a "Review" section and a "Results" section? If so, I would recommend putting into the results section only text that directly relates to Table 1, such that readers that are not interested in a review of existing methods could directly jump to the part where they can learn how to implement the novel OpenSeaDragon based method. I would then also recommend adding more details on how to implement the novel workflow. For instance adding details for how to use the Deepzoom python based approach would be very valuable to make the new workflow accessible to users that do not have a windows computer. In addition, more hands-on information on how to practically share the data with remote collaborators would be very useful (e.g. how to set up a github repository to host the data).

I feel that if the article would be restructured/rewritten along those lines, it could be easier to decide how much and where details about other methods, such as OME-Zarr based workflows, would need to be mentioned.

Detailed comments:

© 2024. Published by The Company of Biologists under the terms of the Creative Commons Attribution License (https://creativecommons.org/licenses/by/4.0/).

Line 99ff:
Thank you for adding the discussion of the OME-Zarr based solution, starting at line 437. I however think that already here in the introduction part it should at least be mentioned. Maybe you could add a sentence like: "Another relevant interactive solution is based on the new OME-Zarr image file format (Moore et al.); this will be discussed in more detail further below."

Line 122: "without the need for a dedicated software or format." I don't think that this is a fair assessment, because the current instructions need the (dedicated) Microsoft ICE msi software and the raw data is converted to the (dedicated) DZI format. This is, in my view, as complex and arguable even less straightforward than, e.g., using the Fiji/ImageJ software (File › Save As › HDF5/N5/Zarr/OME-NGFF …) to convert the data to OME-Zarr format. In addition, maybe even easier than Fiji, one can create OME-Zarr using this tool: **URL**

Line 378: As a citation for the Fiji plugin MoBIE, this dedicated publication seeme more suitable than Vergara et al.: **URL**

Line 456: "format itself could be implemented but the visualisation it is based on repositories"
*I don't think this statement is correct like this. There are no repositories needed for the visualisation. As mentioned in my first review, on can construct an URL (see below) uses the open-access neuroglancer (***URL** *) visualisation tool to open a data set (***URL***) that is hosted on a publicly accessible S3 server at EMBL. Someone just happened to put the data there and there are no curated repositories involved at all. Anyone with access to an S3 object store at their institution or, e.g., at Amazon can put any data there and can use any of the various open-access visualisation tools mentioned in* **URL** *to view the data, without the need for any curation or review. For sensitive data some S3 object stores also offer secret/public key protected access (***URL).**

Neuroglancer URL to visualise a S3 hosted OME-Zarr image: **URL**

<u>Detailed answer to the Reviewer:</u>

I am very grateful to the reviewer for the thorough review of the manuscript. Addressing the following comment a sentence mentioning OME-Zarr was added to the introduction (line 95-98) and the following sentences were adapted accordingly (line 99-103).
"*Line 99ff: Thank you for adding the discussion of the OME-Zarr based solution, starting at line 437. I however think that already here in the introduction part it should at least be mentioned. Maybe you could add a sentence like: "Another relevant interactive solution is based on the new OME-Zarr image file format (Moore et al.); this will be discussed in more detail further below.*""

I would also like to thank the reviewer for the understanding of my naivety towards OME-Zarr and high-end data viewer technology as well as the relevance of its details for our proposed solution. In fact, the complexity of the solutions aiming on finding / generating a universally compatible file format and meeting all the requirements for multimodal data led us to seek for a custom solution with simplicity as the main goal. Existing solutions offering high compatibility and the necessary functionality for multimodal data can quickly become intimidating and complicated to understand - especially for biologists with little or no affinity for technology or coding. I have to admit that, due to its overwhelming set of options, the Neuroglancer interface is one of these examples for me personally.
Our Openseadragon based approach is frequently used in a variety of projects due to its intuitive (to non-IT experts) and very simple interface, the use of common file types and the ease of implementation. This manuscript is intended to make it more readily available to the (electron) microscopy community and imaging scientists rather than image analysis experts and data scientists. One strength of our proposed solution is the wide file format compatibility of the selected software tools allowing users to work with already known file formats and pre-existing files or workflows.
The formulation regarding file formats in the manuscript was obviously misleading as pointed out by the reviewer in the following comment:
"*Line 122: "without the need for a dedicated software or format." I don't think that this is a fair assessment, because the current instructions need the (dedicated) Microsoft ICE msi software and*

© 2024. Published by The Company of Biologists under the terms of the Creative Commons Attribution License (https://creativecommons.org/licenses/by/4.0/).

the raw data is converted to the (dedicated) DZI format. This is, in my view, as complex and arguable even less straightforward than, e.g., using the Fiji/ImageJ software (File › Save As › HDF5/N5/Zarr/OME-NGFF …) to convert the data to OME-Zarr format. In addition, maybe even easier than Fiji, one can create OME-Zarr using this tool: **URL**"
The respective text passage (line 113-115) was changed to: "The solution is flexible in its adaptation and can be self-hosted (e.g. internally at an institution) allowing for full offline access for sensitive data, it doesn't require a single dedicated file format and is compatible with a wide range of commonly used file formats." Although it is true that the "simplest" solution proposed is using Microsoft ICE and the DZI format, we emphasise that a variety of software (also summarised in Table S1) can be used for each task and give relevant examples; see e.g.: line 196-197, 204-205, 208-209, 211-214, 219-220, 224-230, 480-484. This overcomes the limitation to a dedicated single tool or file format – also openseadragon itself supports a wide range of formats even plain image files are possible (with performance limitations).
In this regard addressing:
"*For instance adding details for how to use the Deepzoom python based approach would be very valuable to make the new workflow accessible to users that do not have a windows computer.*"
The options mentioned and the overview in Table S1 also include (several) Microsoft Windows - independent solutions and command line tools that are compatible with most operating systems and based on python. Providing detailed step-by-step instructions for each tool or combination of tools would however, likely result in a complex, difficult-to-navigate guide and, in our opinion, is unlikely to aid implementation. For python tools in particular, this also seems unnecessary as python users typically have basic understanding of command line tools and the tools themselves usually contain detailed documentation / instructions. Furthermore, many of these tools only require a single command (e.g. for deepzoom.py this would be "./Imagefilename-dzi.py" executed in the folder containing the source image file). The given detailed examples in the manuscript are rather intended for users without any prior experience or preferred tool and different versions with different complexity are also available in the supplementary data for inexperienced users with advanced requirements.

Regarding hosting and following the comment: "*In addition, more hands-on information on how to practically share the data with remote collaborators would be very useful (e.g. how to set up a github repository to host the data).*", also here the wide range of options and potential services would not allow to explain a reasonable set of approaches in our opinion. However as before we have preferred the simplest options like hosting on a local machine with subsequent visualisation via a local browser, hosting on a simple server on the intranet and browning locally via IP:port, or uploading to a simple hosting service supporting html. (line 121-123) Typically, the local IT can help set up solutions that are compatible with the respective system for hosting via the intranet, as the requirements can be very different. For the general practical aspect of sharing data, URLs and QR codes are the only options we consider besides implementation in online versions of publications, these options are all represented in the manuscript. (line 352-354, 443-445, 561-564)
Further addressing: "*As mentioned in my first review, on can construct an URL (see below) uses the open-access neuroglancer (***URL** *) visualisation tool to open a data set (***URL***) that is hosted on a publicly accessible S3 server at EMBL. Someone just happened to put the data there and there are no curated repositories involved at all. Anyone with access to an S3 object store at their institution or, e.g., at Amazon can put any data there and can use any of the various open-access visualisation tools mentioned in* **URL** *to view the data, without the need for any curation or review.*"
More advanced hosting solutions or commercial solutions have been avoided throughout the manuscript, and while they can certainly provide additional functionality and convenience, in our opinion they do not add to the simplicity and ease of implementation and are often not necessary. The same argument would, in our opinion, also apply to S3 object stores - apart from the fact that they are available both commercially and at EMBL as well as in some institutions, these are not usual facilities. The additional functionality compared to a simple web server is furthermore, in our opinion, not useful when it comes to simple 2D EM datasets. However, for data with a considerably high traffic, additional functionality such as advanced analyses or immense datasets like volume-EM data such approach would certainly be a good solution. Hence hosting as S3 object and visualisation via Neuroglancer is now mentioned to the manuscript (line263-268) as well as a basic paragraph addressing hosting (line 254-268) and specific exemplary instructions for hosting on GitHub PAGES (point 10ff [line 533-541] in the Materials and Methods section [earlier Table1]). Regarding data protection: "*For sensitive data some S3 object stores also offer secret/public key protected access*

© 2024. Published by The Company of Biologists under the terms of the Creative Commons Attribution License (https://creativecommons.org/licenses/by/4.0/).

*(***URL***).*" In terms of security and privacy for confidential or patient data, it is fair to say that a fully offline solution is generally the more secure option and hence preferred solution compared to an online or even cloud-hosted service even when encrypted.
The wrong claims regarding the necessity of curated datasets or review processes for OME-Zarr repositories ("*Line 456: "format itself could be implemented but the visualisation it is based on repositories" I don't think this statement is correct like this. There are no repositories needed for the visualisation.*") have been removed from the text and/or altered (line 464-476)

"*Line 378: As a citation for the Fiji plugin MoBIE, this dedicated publication seeme more suitable than Vergara et al.:* **URL**"
Veragara et al. 2021 was used as MoBIE was developed and first implemented in this publication, however, Pape et al. 2023 is now added to the references. (line 387)

"*However I could not find any concrete "result" in a sense of a concrete workflow that could be implemented; rather this section reviews some existing tools that are related to the stitching of image data.*" While image stitching is common practice and often even implemented in the acquisition software it was surprising that the use of irregular lines, common in the older analogue methodology is rarely used in modern approaches. It is not a true novelty or "result" in this way but seems like an almost forgotten method and hence worth the emphasis. This aspect is not a key requirement, but it is nevertheless a helpful addition to the downstream methodology as it strongly influences the outcome. Therefore, in our opinion, it can be considered part of the workflow and separating it is unlikely to be useful for understanding the approach. In a way the "*mix of a review of existing methods and concrete recommendations what to do*" seems to us to be a sensible approach that provides an overview of methodological options and possible approaches in context of our suggested solution. Consequently, we do not favour separating the "review" and the novel aspects of the manuscript - these are closely interwoven and are now presented in the logic of the processing steps, which we believe is advantageous for usability and application. In addition, experienced users can see alternative options and approaches at each step of the process within the referenced tools, and familiar solutions can replace steps that we have chosen to prioritise in the manuscript, which in turn can lead to a higher likelihood of implementation. For an overview and summary, Table S1 is intended to provide users with a quick and uncomplicated overview; unfortunately, it could not be included in the main text.

Third decision letter

MS ID#: JOCES/2024/262198

MS TITLE: Expanding the field of view: A simple approach for interactive visualisation of electron microscopy data.

AUTHORS: Jens Wohlmann
ARTICLE TYPE: Tools and Resources

I am happy to tell you that your manuscript has been accepted for publication in Journal of Cell Science, pending standard publication integrity checks.

© 2024. Published by The Company of Biologists under the terms of the Creative Commons Attribution License (https://creativecommons.org/licenses/by/4.0/).
